# Impact of Antioxidant-Rich Whole Foods or Supplements on Skin Health: A Systematic Review and Meta-Analysis of Preclinical and Clinical Studies

**DOI:** 10.3390/antiox15030301

**Published:** 2026-02-27

**Authors:** Yuxin Liang, Yujing Xu, Jung Eun Kim

**Affiliations:** 1 Department of Food Science and Technology, National University of Singapore, Singapore 117543, Singapore; e1124903@u.nus.edu (Y.L.); yujingxu@u.nus.edu (Y.X.); 2 Bezos Centre for Sustainable Protein, National University of Singapore, Singapore 117542, Singapore

**Keywords:** dietary antioxidant, atopic dermatitis, skin aging, psoriasis, acne, oxidative stress

## Abstract

Background: Antioxidant supplements have been reported to confer benefits for skin health; however, these effects remain inconclusive and lack systematic evaluation. Methods: This systematic review and meta-analysis assessed the impact of antioxidant-rich whole foods or supplements on various skin health outcomes by compiling data from five databases, including 94 eligible preclinical and clinical studies. Results: The intervention improved overall skin health in preclinical studies, as evidenced by increased skin hydration (Hedges’ g = 1.75, 95% CI [1.31; 2.20]) and hyaluronic acid, decreased trans-epidermal water loss (TEWL) (Hedges’ g = −2.15, 95% CI [−3.17; −1.13]), epidermal thickness (Hedges’ g = −2.59, 95% CI [−3.28; −1.89]), wrinkle formation, and dermatitis scores, alongside changes in inflammatory cytokines and Immunoglobulin E (IgE) levels. As for clinical studies, the intervention increased skin hydration (MD = 2.12, 95% CI [1.02; 3.21]) while decreased TEWL (MD = −0.68, 95% CI [−1.21; −0.16]). Additionally, changes in skin density, epidermal thickness, minimal erythema dose (MED), SCORing Atopic Dermatitis (SCORAD) and the Dermatitis Life Quality Index (DLQI) further support overall improvements for skin health. Conclusions: Antioxidant-rich whole foods or supplements intake improved overall skin health and skin disorder conditions. The magnitude of benefit may vary according to the type of antioxidant and the duration of intervention.

## 1. Introduction

As the most extensive organ of the human body, the skin functions as a vital interface against environmental insults, regulating temperature, preventing dehydration, and playing a crucial role in immune defense [1]. However, skin diseases such as atopic dermatitis (AD), psoriasis, acne vulgaris, rosacea, and even skin aging may weaken the function of the skin. These skin disorders are highly prevalent worldwide and have been increasingly recognized as significant public health concerns. Beyond cosmetic concerns, these conditions can lead to psychological burden, manifesting as disrupted sleep patterns, heightened anxiety, and depressive symptoms [2].

Skin health is influenced by a complex interplay of intrinsic factors, such as genetics, aging, hormonal changes, and metabolic status, and extrinsic factors like ultraviolet (UV) radiation, air pollution, psychological stress, and dietary patterns [3,4,5,6,7,8]. Oxidative stress is a key component that explains the damaging effects of the aforementioned factors on skin. Reactive oxygen species (ROS) can overwhelm the skin’s antioxidant defense systems, leading to inflammation, lipid peroxidation, DNA damage, and disruption of the skin barrier [9,10,11,12,13,14,15]. This process contributes to chronic inflammatory skin conditions such as AD, psoriasis, and acne through the nuclear factor kappa B (NF-κB) signaling axis and establishes a self-perpetuating cycle of oxidative damage [16,17,18,19,20].

Conventional treatments, including UV phototherapy, topical corticosteroids, biologic agents, and isotretinoin, remain central to clinical management. However, these treatments are associated with adverse effects, particularly with prolonged use [21,22,23,24]. Consequently, interest in safer, more sustainable strategies such as dietary antioxidants has grown, which may help mitigate oxidative damage and support skin health [11,25,26]. Antioxidants from whole foods and dietary supplements have demonstrated the ability to neutralize ROS, reduce inflammation, and enhance endogenous antioxidant defense systems. These properties may further improve skin barrier function, support collagen synthesis, increase hydration, and alleviate inflammation-associated skin conditions [25,27,28,29,30,31,32,33]. Clinical studies have also shown improvements in skin hydration and photoprotection, as well as reductions in symptom severity in AD and psoriasis patients following supplementation with astaxanthin, docosahexaenoic acid, or vitamin D [34,35,36].

Despite these promising findings, current research remains fragmented. Most studies focus on isolated antioxidant supplements, leaving a gap in evidence regarding antioxidant-rich whole food interventions and comparative outcomes across various skin conditions. Furthermore, results remain inconsistent due to variations in food sources, supplement types, and intervention durations. For example, a study that used astaxanthin as a supplement showed a reduction in skin hydration [33], while other studies that supplemented with extracts and fatty acids showed increased skin hydration [37,38] during UV-induced skin deterioration in healthy adults. In addition, one study reported a significant increase in skin hydration after 16 weeks of almond consumption, whereas no significant improvement was observed over 8 weeks of consumption [39]. To address these gaps, this systematic review and meta-analysis aimed to evaluate the effects of antioxidant-rich whole foods and dietary supplements on various skin conditions. Evidence from randomized controlled trials and animal studies was synthesized to quantify overall effects and explore potential sources of heterogeneity. We hypothesized that antioxidant intake enhances skin barrier function and reduces symptom severity in skin disorders. Subgroup analyses were further conducted based on antioxidant type (whole food or supplement) and intervention duration.

## 2. Materials and Methods

This review was performed according to the Preferred Reporting Items for Systematic Reviews and Meta-Analysis (PRISMA) guidelines [40], including identification, screening, eligibility and inclusion. The current review protocol has also been registered on PROSPERO (ID: CRD420251022948).

### 2.1. Search Strategy and Selection Criteria

A systematic literature review was performed across five primary databases—PubMed, Embase, Web of Science, the Cochrane Library, and CINAHL—with an initial search date of 1 June 2024 and a subsequent update in March 2025. Two independent investigators (Y.L. and Y.X.) identified relevant studies based on a predefined PICOS framework (Table 1). Specific search strategies and database-specific filters are detailed in Supplementary Table S1.

Inclusion criteria are as follows: (1) RCTs for clinical studies with population age above 24 months; (2) preclinical studies; (3) intervention group receiving oral antioxidant-rich whole foods or supplements, and control group receiving placebo or not receiving oral antioxidant-rich whole foods or supplements; (4) reporting at least one of the outcomes: Dermatitis score, Pruritus score, hydration, TEWL, hyaluronic acid, epidermal thickness, wrinkle formation, skin sebum, skin pH, skin density, wrinkle formation, skin elasticity, skin MED, SCORAD, DLQI, Eczema Aera Severity Index (EASI), Psoriasis Area Severity Index (PASI), Body Surface activity (BSA), Disease Activity Score 28—C-Reactive Protein (DAS28-CRP), and Acne count. Studies that lack necessary information were excluded.

Initial records identified through the database search were managed using EndNote 21 software to systematically eliminate duplicates. Two investigators (Y.L. and Y.X.) then independently assessed the remaining literature by screening titles and abstracts for alignment with the study objectives. Following this preliminary phase, full-text versions of potentially eligible papers were retrieved for a rigorous final evaluation. Any disagreements regarding study inclusion during the screening process were adjudicated through discussion with a third senior reviewer (J.E.K.).

### 2.2. Data Extraction

Primary (Y.L.) and secondary (Y.X.) reviewers were assigned to abstract and full-text screening against inclusion and exclusion criteria, followed by data extraction and risk of bias assessment. From each paper, the following information was extracted. Preclinical studies: Author(s), year of publication, country in which the study was conducted, number of participants, study design, intervention duration, animal model, age, treatment groups, gender, antioxidant-rich whole foods or supplements intake and food source. For clinical study, apart from information mentioned for preclinical studies, skin type of participants was extracted from each paper. Antioxidant-rich whole foods or supplements were categorized into multivitamins, extracts, polyphenols, fatty acids, carotenoids, fruits, vegetables, nuts, etc. Secondary outcomes including biomarkers of oxidative stress: Malondialdehyde (MDA), Superoxide Dismutase (SOD), Catalase (CAT), Glutathione Peroxidase (GPx); inflammation: interleukin-1 beta (IL-1β), interleukin-6 (IL-6), Tumor Necrosis Factor alpha (TNF-α); allergen: IgE; skin structural: Collagen type 1; antioxidant status: blood antioxidant level and total antioxidant capacity (TAC) were also extracted.

In accordance with *Cochrane Handbook* guidelines (Section 23.2) [41], crossover trials were treated as parallel-group trials due to a lack of reported correlation coefficients; data were analyzed as parallel trials by incorporating the total number of participants in both the treatment and control cohorts. To maintain data integrity, the use of adequate washout periods was verified across all included crossover studies to minimize potential carry-over effects. Furthermore, in studies featuring multiple intervention arms, each treatment group was evaluated independently against the corresponding control group to ensure distinct comparisons.

### 2.3. Risk of Bias Assessment

Two independent reviewers (Y.L. and Y.X.) evaluated the methodological quality of all included studies. For preclinical studies, the Systematic Review Centre for Laboratory Animal Experimentation (SYRCLE)’s risk of bias tool was utilized to assess potential biases related to randomization, missing data, selective reporting and other potential sources [42]. Clinical studies were appraised using a modified Cochrane Risk of Bias tool (RoB 2), which examined domains such as the randomization process, deviations from intended interventions, missing data, and outcome measurement [43]. Specifically for crossover clinical trials, an additional domain was included to account for carryover and period effects. Any assessment conflicts were reconciled through consultation with a third reviewer (J.E.K.). Furthermore, publication bias was quantitatively and qualitatively scrutinized using Egger’s regression test and visual inspection of funnel plots, respectively. A bias-aware sensitivity test was also conducted for the publication bias for the selected studies.

### 2.4. Data Synthesis and Statistical Analysis

When necessary, mean and standard deviation (SD) values were estimated from reported median and interquartile ranges. To determine effect sizes, the absolute mean difference (MD) with its corresponding standard error was utilized for clinical outcomes. For preclinical data, Hedges’ g was calculated to account for potential small-sample size bias. All statistical procedures adhered to the guidelines in the *Cochrane Handbook* [41].

All meta-analytic procedures were executed using R software (version 4.5.2), utilizing the *metagen* function to calculate the pooled impact of antioxidant-rich whole foods and supplements on skin health. We expressed the combined effect as mean differences (MDs) alongside their corresponding 95% confidence intervals (CIs). Inter-study heterogeneity was quantified via the I^2^ statistic, where values exceeding 50% were considered indicative of significant variance. Accordingly, a random-effects model was employed to synthesize data with substantial heterogeneity (I^2^ > 50%), while a fixed-effect model was reserved for cases of low heterogeneity (I^2^ ≤ 50%). To investigate potential drivers of variance, we performed a ‘leave-one-out’ sensitivity analysis using the *metafor* package. Additionally, predefined subgroup analyses were conducted based on intervention length and antioxidant categories for both preclinical and clinical cohorts. Statistical significance for all tests was established at a two-tailed *p* < 0.05.

## 3. Results

### 3.1. Study Selection Progress

**Figure 1:**
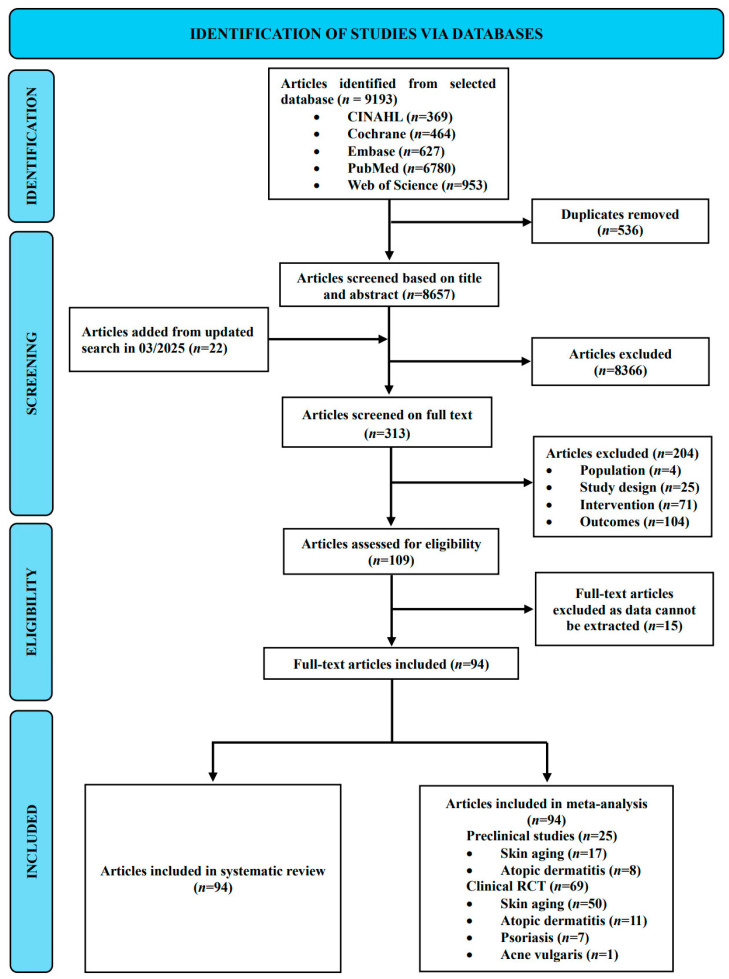
PRISMA flowchart for the systematic review and meta-analysis.

Systematic searching was conducted across five databases: CINAHL (*n* = 369), Cochrane (*n* = 464), Embase (*n* = 627), PubMed (*n* = 6780), and Web of Science (*n* = 953), yielding a total of 9193 studies. After removing 536 duplicate studies, 8657 studies remained for title and abstract screening. An updated search yielded an additional 22 investigations, while 8366 records were dismissed following a preliminary review of titles and abstracts. This left 313 studies for a comprehensive full-text assessment. During this phase, 204 papers were excluded for failing to satisfy the inclusion criteria: specifically, 104 reported irrelevant outcomes, 71 utilized ineligible interventions, 25 employed incorrect study designs, and four targeted inappropriate populations. Furthermore, 15 studies were omitted due to a lack of extractable data. Ultimately, 94 studies were incorporated into the systematic review and meta-analysis, comprising 25 preclinical models and 69 clinical trials. The flow diagram for the strategy of this study is shown in Figure 1.

### 3.2. Study Characteristics

**Figure 2:**
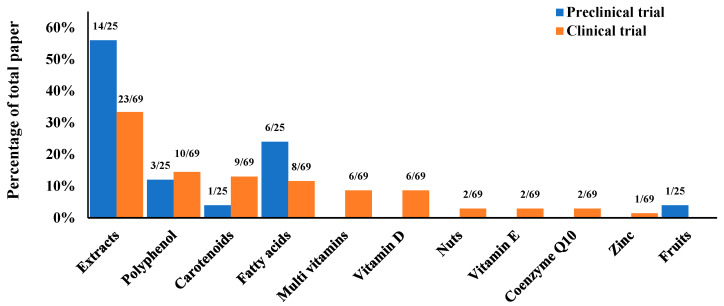
Percentage of total papers reporting on antioxidant-rich whole foods or supplements from preclinical and clinical trials.

The detailed characteristics of all included studies are summarized in Supplementary Tables S2–S4. Figure 2 illustrates the distribution of intervention types across the included studies. A total of 94 articles were identified and categorized, comprising 25 preclinical studies and 69 clinical trials. Among the preclinical studies (*n* = 25), the majority of articles focused on Extracts (14/25, 56%), followed by Fatty acids (6/25) and Polyphenols (3/25). In the clinical trials (*n* = 69), Extracts were also the most frequently investigated intervention (23/69, 33%). This was followed by Polyphenols (10/69), Carotenoids (9/69), and Fatty acids (8/69). Notably, interventions such as Multivitamins (6/69) and Vitamin D (6/69) were exclusively reported in clinical trials. A more detailed summary and classification of the antioxidant-rich whole foods or supplements type can be found in Supplementary Table S5.

For the analyses of the skin aging and AD conditions in animal models, 25 studies with 55 comparisons were included [30,31,44,45,46,47,48,49,50,51,52,53,54,55,56,57,58,59,60,61,62,63,64,65,66]. Among these, 17 studies with 34 comparisons examined the effects of antioxidant-rich whole foods or supplements on UV-induced skin aging, assessing both phenotypic and genotypic outcomes. The other 8 studies with 21 comparisons investigated AD animal models, including 2,4-dinitrochlorobenzene (DNCB)-induced AD mouse model and canine subjects with perennial AD. Of the 55 total comparisons, interventions primarily consisted of plant or fruit extracts (*n* = 31), followed by fatty acids (*n* = 12), polyphenols (*n* = 7), and various carotenoids or whole fruits. Study durations ranged from 2 to 14 weeks, with outcomes measured via skin barrier function, oxidative stress, inflammation, structural integrity, and allergy-related biomarkers.

With respect to controlling skin health on skin aging population, 50 studies (58 comparisons) with a total of 3175 participants were included [33,38,39,67,68,69,70,71,72,73,74,75,76,77,78,79,80,81,82,83,84,85,86,87,88,89,90,91,92,93,94,95,96,97,98,99,100,101,102,103,104,105,106,107,108,109,110,111,112,113]. Among them, 391 participants were healthy postmenopausal females with aging skin, and the remainder were healthy adults with aging skin. Across the 58 comparisons identified, interventions included plant extracts (*n* = 21), carotenoids (*n* = 12), multivitamins (*n* = 7), and polyphenols (*n* = 7), with a smaller subset investigating fatty acids (*n* = 6), nuts (*n* = 3), and coenzyme Q10 (*n* = 2). Study durations spanned 4 to 24 weeks. Regarding inflammatory skin conditions, 19 studies (encompassing 20 comparisons and 1131 participants) were included in the analysis [34,35,36,114,115,116,117,118,119,120,121,122,123,124,125,126,127,128,129]. Among them, 65 participants were patients with psoriatic arthritis, 291 were patients with mild-moderate psoriasis, 751 were patients with mild-moderate atopic dermatitis and 24 were patients suffering from acne vulgaris. Across these 20 comparisons, interventions were diverse: vitamin D supplements were the most frequent (*n* = 6), followed by fatty acids (*n* = 4) and plant extracts (*n* = 4). Additional interventions included polyphenols (*n* = 2), vitamin E (*n* = 2), and single comparisons involving coenzyme Q10 and carotenoids. Study durations varied significantly, ranging from 2 weeks to 12 months. Overall, clinical studies investigated skin barrier function, blood antioxidant levels, antioxidant enzyme levels and oxidative stress biomarkers for skin aging. As for skin disorder conditions, eczema severity, psoriasis severity, and acne severity were measured, while dermatology life quality and allergy biomarkers were also tested during these studies.

### 3.3. Meta-Analysis Results from Preclinical Studies

Table 2 shows the overall effect of antioxidant-rich whole foods or supplements on preclinical skin health. Skin hydration (Hedges’ g = 1.75, 95% CI [1.31; 2.20]) and hyaluronic acid (Hedges’ g = 2.05, 95% CI [0.93; 3.17]) increased, while TEWL (Hedges’ g = −2.15, 95% CI [−3.17; −1.13]), epidermal thickness (Hedges’ g = −2.59, 95% CI [−3.28; −1.89]), and wrinkle formation (Hedges’ g = −4.28, 95% CI [−5.79; −2.76]) were all significantly reduced after treatments of antioxidant-rich whole foods or supplements. However, no changes were observed for pruritus score. For AD model, the dermatitis severity (Hedges’ g = −2.55, 95% CI [−4.43; −0.67]) also decreased significantly. Secondary outcomes indirectly reflected the improvement for AD severity, and antioxidant enzymes, including SOD, CAT and GPx levels, were all significantly increased, while anti-inflammatory cytokines such as interleukin-1β (IL-1β), interleukin-6 (IL-6), and tumor necrosis factor-α (TNF-α) were all decreased. Allergy biomarker IgE levels also significantly decreased (Supplementary Table S6).

**Figure 3:**
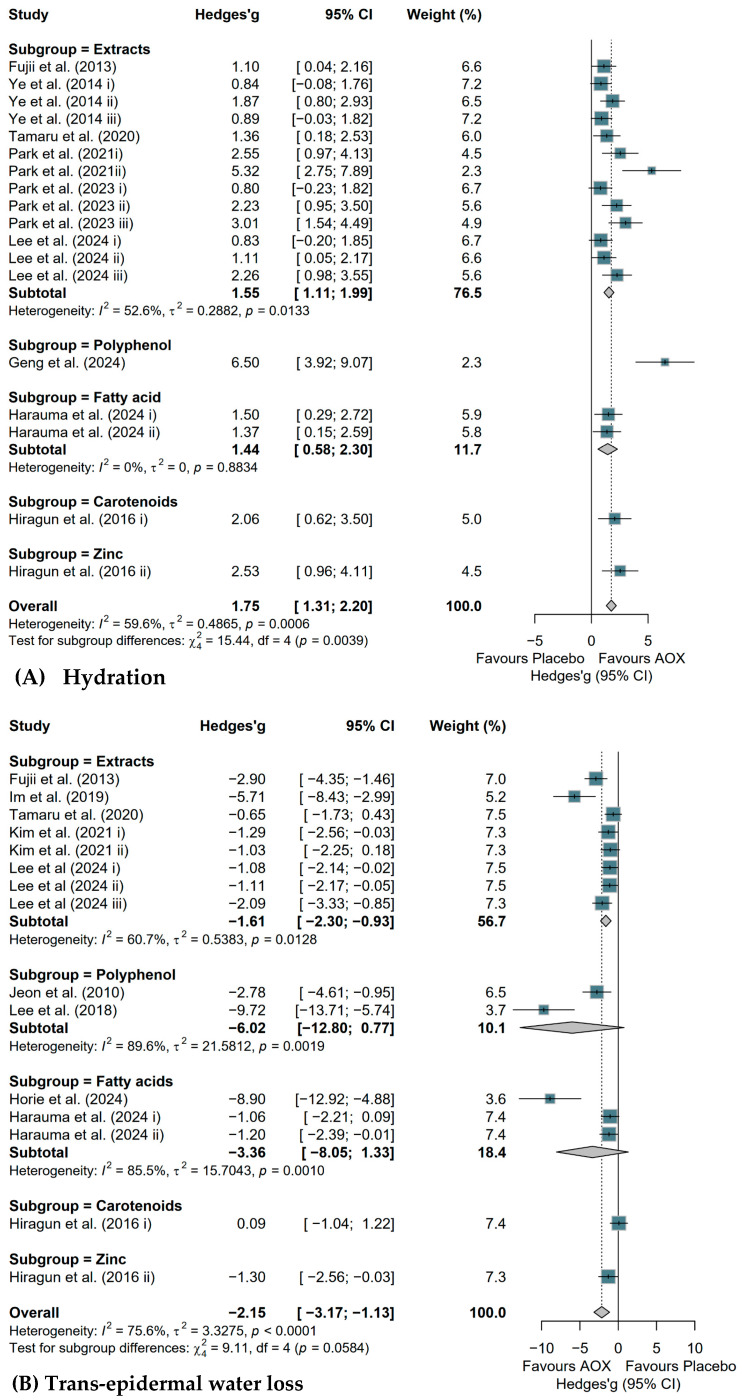

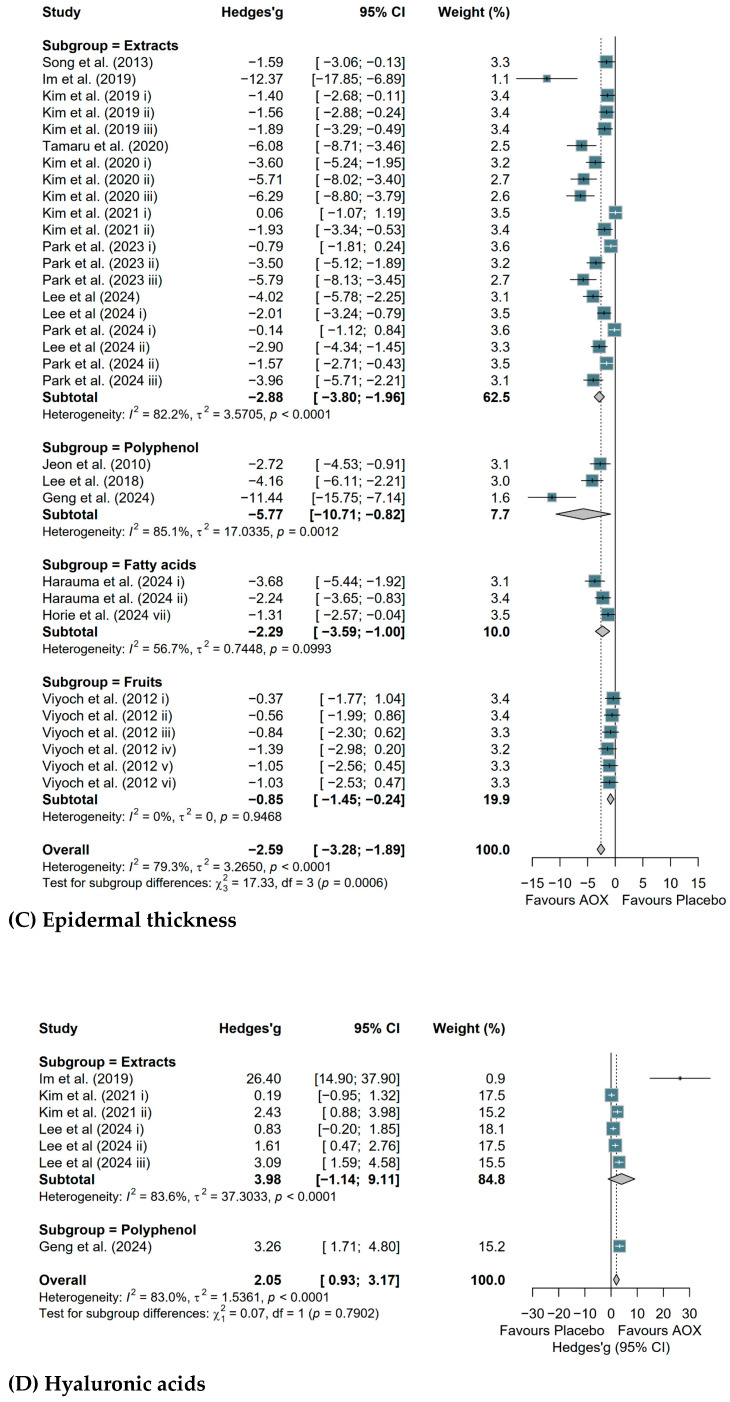

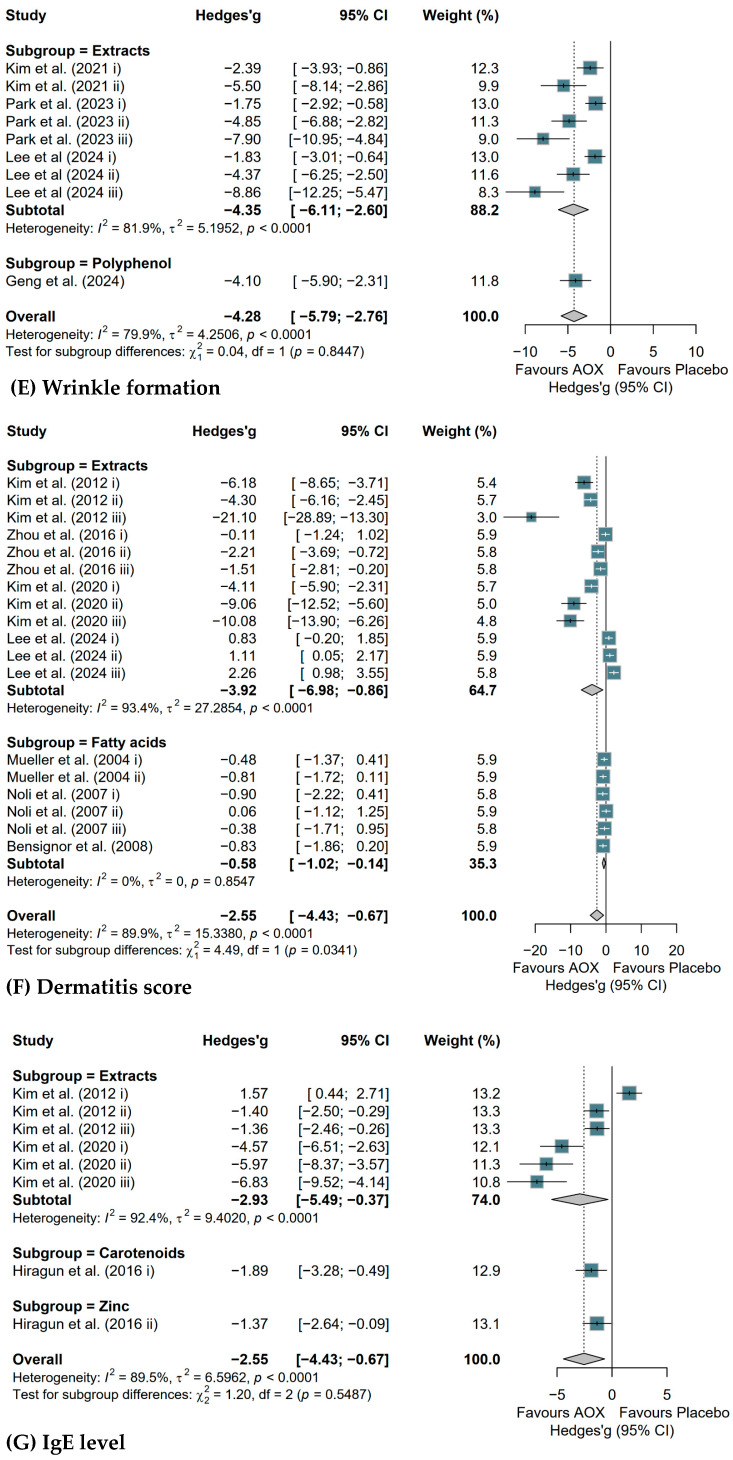
Forest plots illustrating the effects of antioxidant-rich whole foods or supplements (AOX) consumption on animal skin health, with subgroup analysis based on the AOX type of study cohorts, evaluated by meta-analysis. Squares (Dark Teal): Individual study effect sizes; square size is proportional to the study’s weight based on precision and sample size; Diamonds (Light Grey): Pooled effect sizes for subgroups and overall results; the center denotes the point estimate, while the width represents the 95% CI; Vertical Dashed Line: Represents the overall mean effect size across all included studies. The effect of AOX consumption on (**A**) skin hydration in preclinical studies; (**B**) trans-epidermal water loss in preclinical studies; (**C**) epidermal thickness in preclinical studies; (**D**) hyaluronic acid in preclinical studies; (**E**) wrinkle formation in preclinical studies; (**F**) dermatitis score in preclinical studies; (**G**) IgE level in preclinical studies. Studies mentioned in the plots: [30,31,44,45,46,47,48,49,50,51,52,53,54,55,56,57,58,59,60,61,62,63,64,65,66].

Results of subgroup analyses based on antioxidant-rich whole foods or supplement types are shown in Figure 3. Extracts, polyphenols, and fatty acids showed significant improvements in skin barrier functions. Particularly, extracts significantly increased skin hydration (Hedges’ g = 1.55, 95% CI [1.11; 1.99]) (Figure 3A), while significantly decreasing TEWL (Hedges’ g = −1.61, 95% CI [−2.30; −0.93]) (Figure 3B), epidermal thickness (Hedges’ g = −2.88, 95% CI [−3.80; −1.96]) (Figure 3C), wrinkle formation (Hedges’ g = −4.35, 95% CI [−6.11; −2.60]) (Figure 3E), dermatitis scores (Hedges’ g = −3.92, 95% CI [−6.98; −0.86]) (Figure 3F), and IgE levels (Hedges’ g = −2.93, 95% CI [−5.49; −0.37]) (Figure 3G). Polyphenol led to significant reductions in epidermal thickness (Hedges’ g = −5.77, 95% CI [−10.71; −0.82]). Fatty acids showed significant increases in hydration (Hedges’ g = 1.44, 95% CI [0.58; 2.30]) and reductions in both epidermal thickness (Hedges’ g = −2.29, 95% CI [−3.59; −1.00]) and dermatitis scores (Hedges’ g = −0.58, 95% CI [−1.02; −0.14]).

Regarding intervention duration (Supplementary Figure S1), antioxidant-rich whole foods or supplements treatments lasting less than 12 weeks showed overall improvements as evidenced by increasing hydration (Hedges’ g = 1.57, 95% CI [1.22; 6.61]) and hyaluronic acid levels (Hedges’ g = 1.75, 95% CI [0.51; 2.98]), alongside decreasing TEWL (Hedges’ g = −1.48, 95% CI [−2.10; −0.85]), epidermal thickness (Hedges’ g = −3.02, 95% CI [−3.80; −2.24]), wrinkle formation (Hedges’ g = −4.62, 95% CI [−6.90, −2.33], and oxidative stress biomarkers IL-1β (Hedges’ g = −2.48, 95% CI [−3.46; −1.50]), IL-6 (Hedges’ g = −2.31, 95% CI [−3.52; −1.81], and TNF-α mRNA (Hedges’ g = −3.20, 95% CI [−4.51; −1.88]). Although limited, ≥12 weeks subgroup showed improvements in certain parameters including TEWL (Hedges’ g = −4.11, 95% CI [−7.95; −0.27]), epidermal thickness (Hedges’ g = −2.17, 95% CI [−3.45; −0.88]), and wrinkle formation (Hedges’ g = −3.76, 95% CI [−5.47; −2.76]).

### 3.4. Meta-Analysis Results from Clinical Studies

Table 3 shows the effect of antioxidant-rich whole foods or supplements on clinical skin health outcomes. Skin hydration (MD = 2.12, 95% CI [1.02; 3.21]), epidermal thickness (MD = 0.12, 95% CI [0.05; 0.19]), skin density (MD = 0.68, 95% CI [0.32; 1.04]), MED (MD = 21.56, [0.07; 43.04]) were increased while TEWL decreased (MD = −0.68, 95% CI [−1.21; −0.16]) after antioxidant-rich whole foods or supplements intervention. Positive effects on skin AD severity and quality of life were also found, as evidenced by decreasing SCORAD index (MD = −15.16, 95% CI [−29.35; −0.97]) and DLQI (MD = −2.60, 95% CI [−4.98; −0.23]). However, skin elasticity, skin sebum, and PASI did not show any changes.

**Figure 4:**
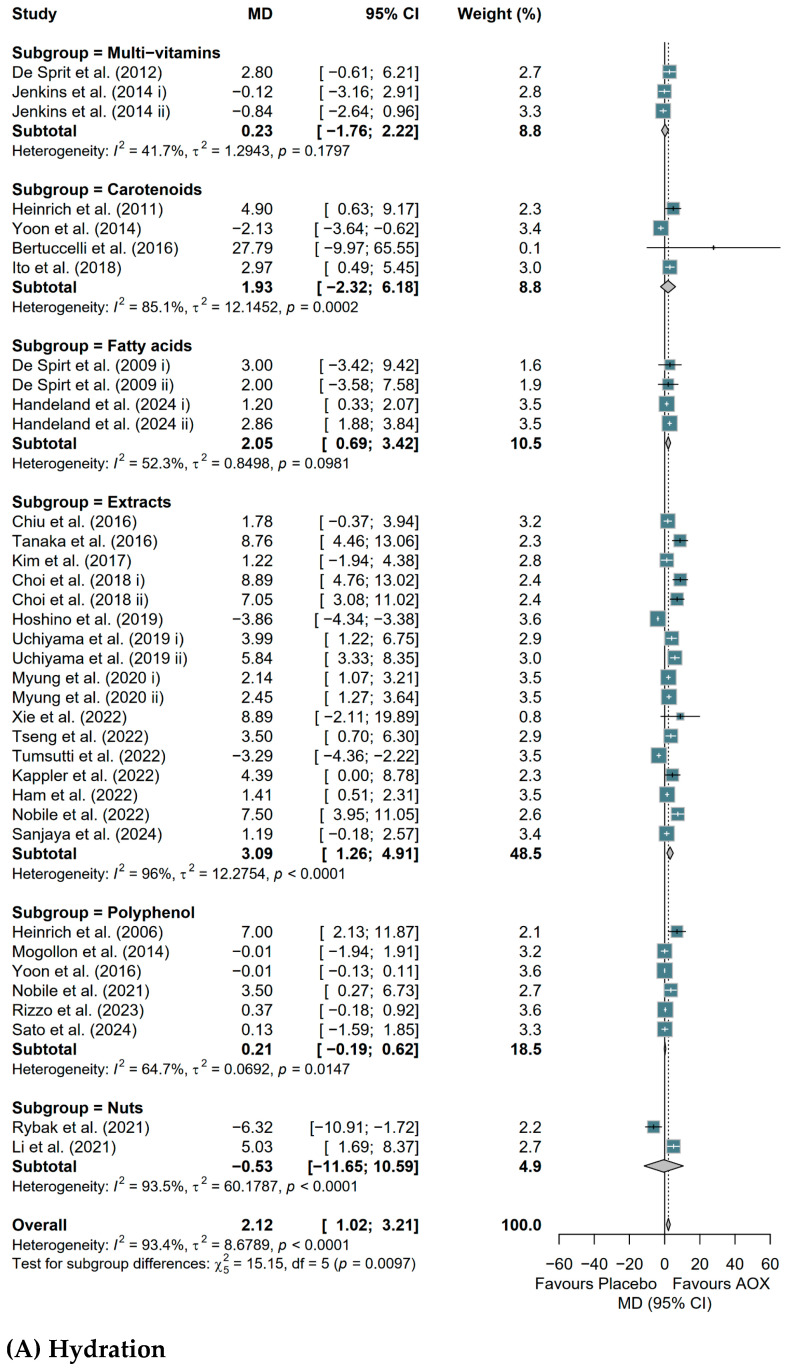

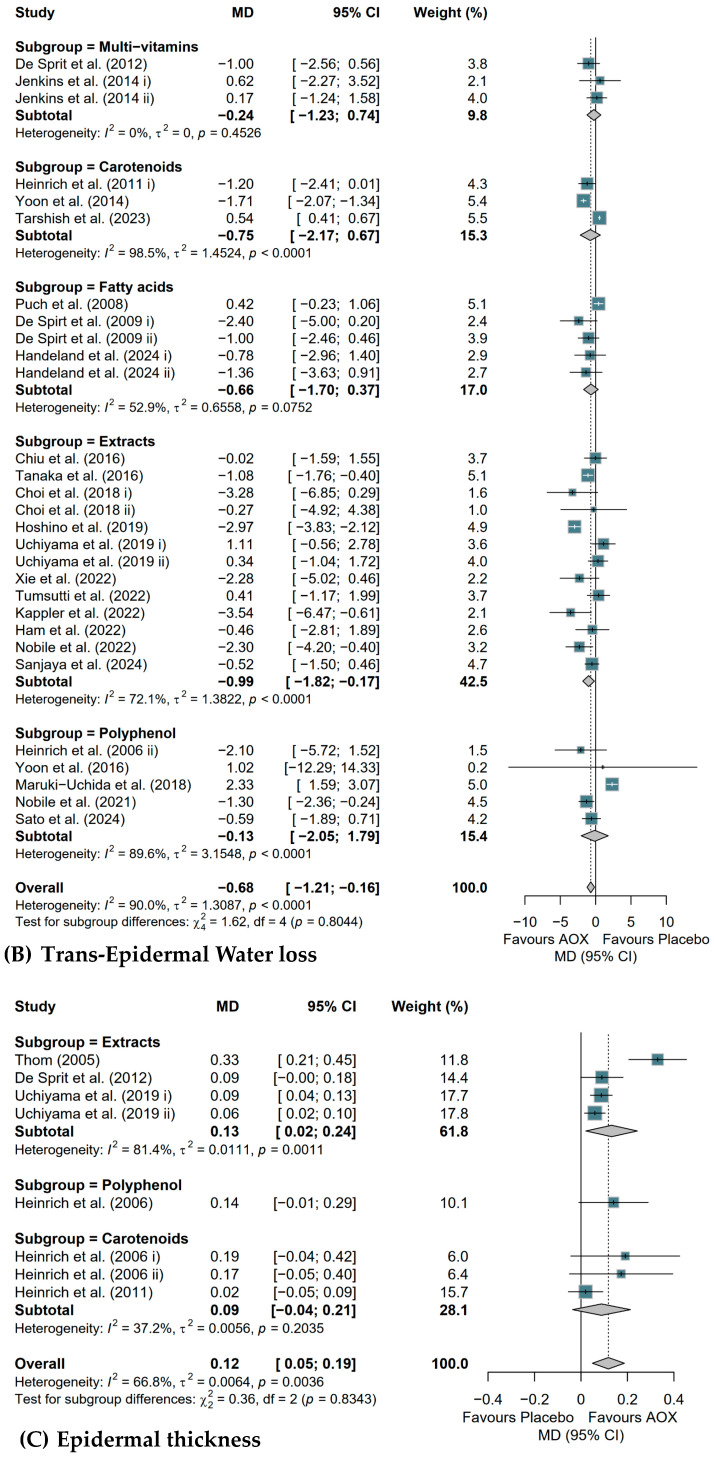

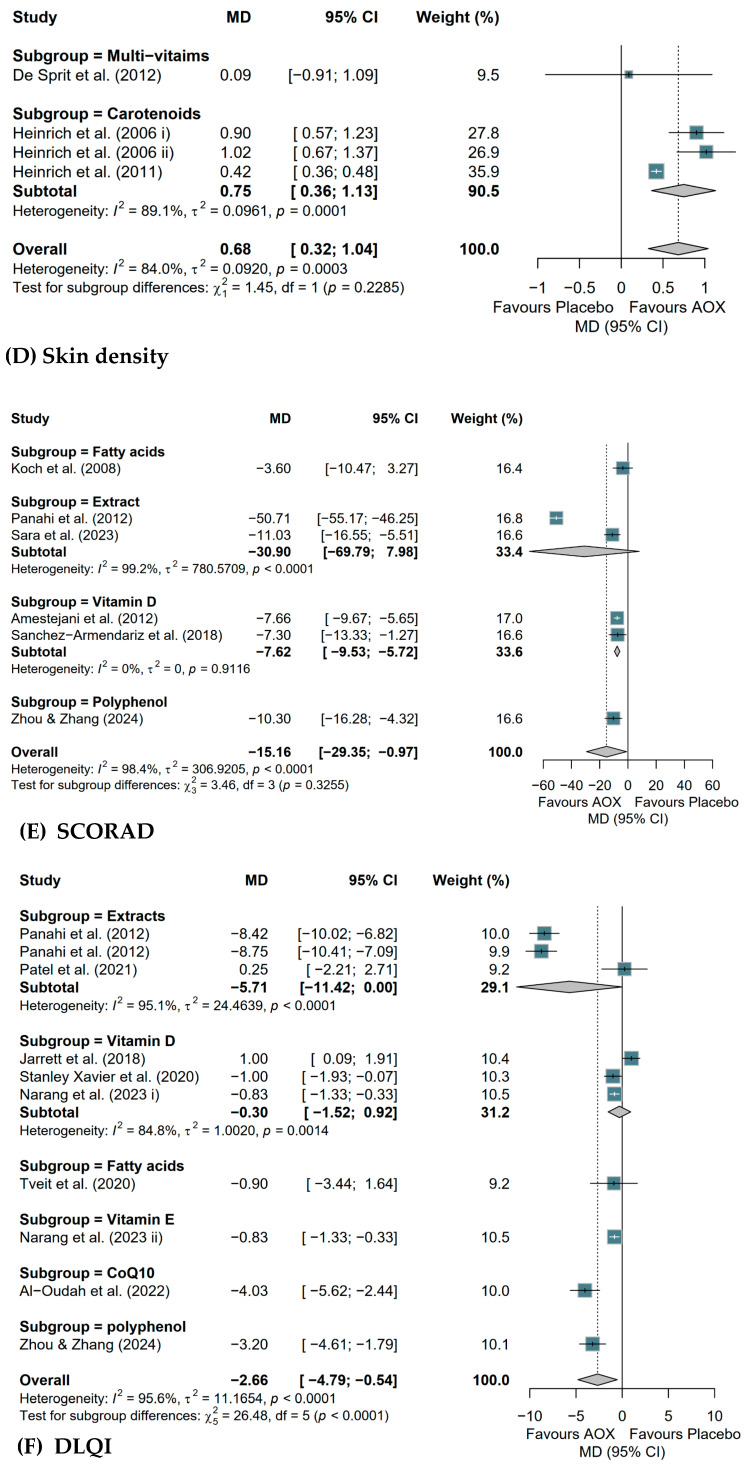

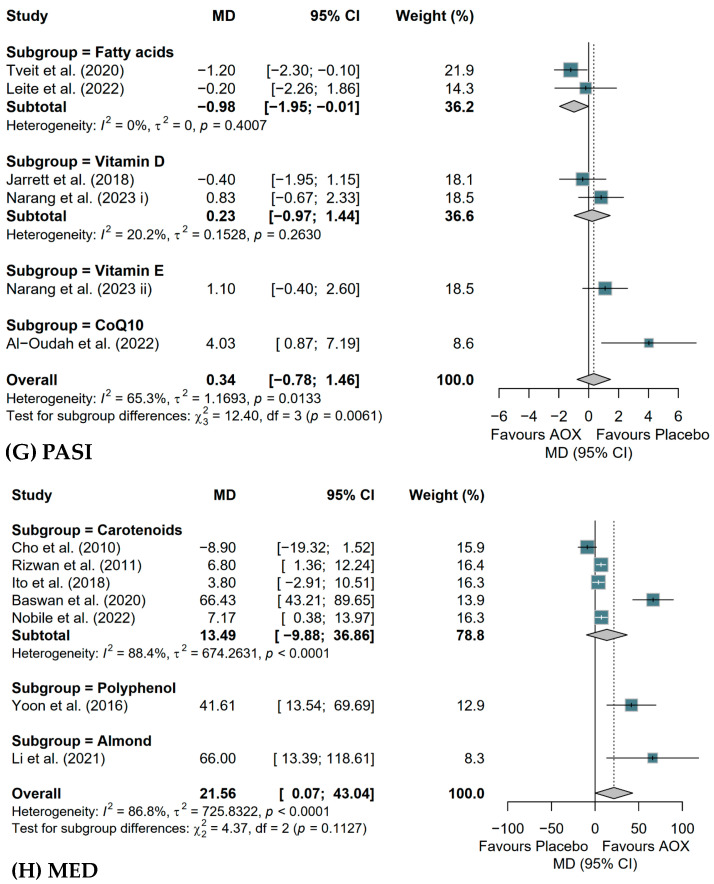
Forest plots of the effects of antioxidant-rich whole foods or supplements (AOX) consumption on human skin health, with subgroup analysis based on the AOX type of study cohorts, evaluated by meta-analysis. Squares (Dark Teal): Individual study effect sizes; square size is proportional to the study’s weight based on precision and sample size; Diamonds (Light Grey): Pooled effect sizes for subgroups and overall results; the center denotes the point estimate, while the width represents the 95% CI; Vertical Dashed Line: Represents the overall mean effect size across all included studies. The effect of AOX consumption on (**A**) skin hydration in clinical studies; (**B**) trans-epidermal water loss in clinical studies; (**C**) epidermal thickness in clinical studies; (**D**) skin elasticity in clinical studies; (**E**) SCORAD in clinical studies; (**F**) DLQI in clinical studies; (**G**) PASI in clinical studies; (**H**) MED in clinical studies. Studies mentioned in the forest plots: [33,34,35,36,38,39,67,68,69,70,71,72,73,74,75,76,77,78,79,80,81,82,83,84,85,86,87,88,89,90,91,92,93,94,95,96,97,98,99,100,101,102,103,104,105,106,107,108,109,110,111,112,113,114,115,116,117,118,119,120,121,122,123,124,125,126,127,128,129].

The result of subgroup analysis by types of antioxidant-rich whole foods or supplements is shown in Figure 4, and different types showed differential responses in different skin outcomes. Extracts significantly improved skin hydration (MD = 3.09, 95% CI [1.26, 4.91]) (Figure 4A) and epidermal thickness (MD = 0.13, 95% CI [0.02, 0.24]) (Figure 4C) and significantly reduced TEWL (MD = −0.99, 95% CI [−1.82, −0.17]) (Figure 4B). Fatty acids showed a positive effect on skin hydration (MD = 2.05, 95% CI [0.69, 3.42]) (Figure 4A) and a reduction in psoriasis severity, as measured by PASI scores (MD = −0.98, 95% CI [−1.95, −0.01]) (Figure 4G). Carotenoids increased skin density (MD = 0.75, 95% CI [0.36, 1.13]) (Figure 4D), and vitamin D supplementation decreased AD severity (MD = −7.62, 95% CI [−9.53, −5.72]) (Figure 4E). There were no effects on DLQI (Figure 4F) or MED (Figure 4H) based on types of antioxidant-rich whole foods or supplements.

Supplementary Figure S2 showed the results of subgroup analysis based on intervention duration. Studies with an intervention period of ≥12 weeks showed overall significant improvements in skin outcomes, while those with an intervention period of <12 weeks did not show such effects. After ≥12 weeks of antioxidant-rich whole foods or supplements intake, there were significant increases in skin hydration (MD = 2.06, 95% CI [0.99, 3.13]), skin elasticity (MD = 0.03, 95% CI [0.01, 0.06]), epidermal thickness (MD = 0.12, 95% CI [0.05, 0.19]), and skin density (MD = 0.68, 95% CI [0.32, 1.04]). Additionally, TEWL (MD = −0.71, 95% CI [−1.30, −0.13]) and SCORAD scores (MD = −8.81, 95% CI [−13.06, −4.56]) were significantly reduced. There was no effect on DLQI based on duration.

### 3.5. Publication Bias and Sensitivity Test

The results of the sensitivity test for the primary outcomes are shown in Supplemental Tables S7–S31. The risk of bias assessments of individual studies is detailed in Supplemental Figures S3 and S4. In preclinical studies, four studies were judged as having some concerns due to insufficient information on randomization, and six studies were rated as high risk due to unexplained missing outcome data (*n* = 5) and selective reporting (*n* = 1). Among clinical studies, most of the studies had some concerns in different domains. In total, 29 studies were considered to have a low risk of bias across all assessed domains. Quantitatively, publication bias was scrutinized via funnel plots and Egger’s regression (Supplemental Figures S5 and S6) for outcomes exceeding ten comparisons. Evidence of significant publication bias was observed for hydration, epidermal thickness, SOD and CAT in preclinical studies, as well as hydration and TEWL in clinical studies (Egger’s test *p* < 0.05). To evaluate the impact of these small-study effects, bias-aware sensitivity analyses were performed for primary outcomes (Supplemental Tables S32–S41). These analyses confirmed that publication bias significantly influenced the pooled effect sizes for hydration, epidermal thickness, and SOD in preclinical studies, alongside hydration, TEWL, skin elasticity, and DLQI in clinical studies (*p* > 0.05).

## 4. Discussion

Beneficial effects of antioxidant-rich whole foods and dietary supplements on skin health have been well examined [28,29,30,31], but this evidence has not been systematically reviewed. By integrating data from both preclinical and clinical studies, this study revealed that regular consumption of antioxidant-rich whole foods and dietary supplements can enhance skin barrier function and alleviate skin disorder symptoms. Subgroup analysis of preclinical and clinical studies further suggests that extracts containing multiple bioactive compounds consistently improve skin barrier function and disease severity.

In animal models, interventions of antioxidant-rich whole foods or supplements enhance skin barrier function by significantly improving skin hydration and hyaluronic acid, and reducing TEWL and wrinkle formation. These enhancements are likely due to the neutralization of ROS by antioxidants, which prevents oxidative damage to skin lipids and proteins, thereby maintaining skin integrity [25]. Notably, an upregulation of antioxidants enzyme expression—such as SOD, CAT, and GPx—was observed, suggesting activation of the nuclear factor erythroid 2–related factor 2 (Nrf2) pathway. Nrf2 serves as a central regulator of the endogenous antioxidant response, thereby restoring the skin’s redox environment and limiting oxidative-mediated tissue damage [130]. Previous studies demonstrate that extracted compounds from plants, such as sulforaphane, curcumin, and turmeric, can increase antioxidant and detoxification capacities by stimulating the Nrf2 pathway in various cell types, including skin cells [131,132]. Beyond direct antioxidant defense, this molecular stabilization likely plays a critical role in modulating the downstream immune response, particularly in inflammatory conditions such as AD. Our findings indicate that antioxidant-induced redox stability leads to a significant suppression of T helper 2 cell (Th2)-dominant inflammation, as evidenced by decreased IgE levels and overall dermatitis scores [133]. This systemic shift is further supported by the reduction in pro-inflammatory cytokines such as IL-1β, IL-6, and TNF-α. Reduced IL-1β and TNF-α are associated with attenuation of keratinocyte hyperproliferation and epidermal thickening, both of which are hallmarks of inflammatory skin disorder [134]. Likewise, decreased IL-6 levels have been linked to the maintenance of barrier integrity by limiting immune cell infiltration and tissue damage [135]. However, pruritus scores did not significantly change. This may be explained by the neuroimmune underlying itch in AD, which involves IL-31, thymic stromal lymphopoietin, and histamine-independent neuronal circuits that may be less responsive to antioxidant modulation [136]. While these immunological changes reduce inflammatory cytokines, the visible rejuvenation of the skin is ultimately realized through distinct structural changes occurring across the epidermal and dermal layers. Improvements in hydration, TEWL, and epidermal thickness are primarily mediated through reducing oxidative stress in keratinocytes and stabilization of epidermal lipid biosynthesis [137]. In contrast, wrinkle reduction and hyaluronic acid formation are largely dependent on dermal remodeling, including collagen and elastin synthesis, matrix metalloproteinase regulation (MMPs), and extracellular matrix (ECM) turnover. Wrinkle reduction depends on ECM turnover. Antioxidants inhibit MMPs, which are enzymes responsible for the degradation of collagen and elastin.t As MMP activity decreases, the rate of new collagen synthesis can eventually outpace degradation, leading to visible wrinkle reduction. While hyaluronic acid can turn over quickly, its sustained accumulation in the dermis relies on the stabilization of the dermal environment. Long-term intake ensures a consistent supplement of precursors and protective antioxidants that prevent hyaluronidases from breaking down the hyaluronic acid [138,139].

Among the various antioxidant-rich interventions, botanical and fruit extracts appeared to be the most effective in modulating skin barrier function, anti-inflammatory markers, and oxidative stress biomarkers. This efficacy is likely attributable to the diverse bioactive profiles of these extracts, as their synergistic blend of phenolics, flavonoids, and vitamins provides a broad spectrum of biological activities that enhance antioxidant defenses and reinforce barrier integrity [66]. Previous studies have reported that extracts from plants or fruits showed a significant improvement in skin barrier function when compared with ascorbic acid only in UV-irradiated mouse skin [44,55]. The polyphenols subgroup showed reductions in TEWL and epidermal thickness in preclinical studies, and this is mainly due to its ability to scavenge ROS in keratinocytes and lipids of the stratum corneum, decreasing the oxidative disruption of epidermal lipids [140]. Similarly, fatty acids contributed to improvements in skin barrier function outcomes in the UV-irradiated model, and this can be explained by their structural role in stratum corneum lipids and protection against oxidative damage [141]. Beyond these shared effects, fatty acids additionally reduced dermatitis severity scores in AD models. This improvement is likely due to the combined restoration of barrier lipid composition and modulation of Th2-driven inflammation, underscoring their dual role in barrier support and immune regulation [141].

Meta-analysis results from clinical studies suggest that antioxidant-rich whole foods or supplements can positively modulate various aspects of skin health, including skin hydration, skin barrier, and skin density. These effects are potentially mediated by enhanced protection against oxidative stress. Although results of biological markers were not feasible due to the limited studies, mechanistic insights into anti-inflammation and antioxidant properties of antioxidant food or supplements from preclinical studies support the observed clinical outcomes. Additionally, a previous study reported that a 4-week curcumin supplementation in AD patients can significantly increase serum levels of antioxidant enzymes, including SOD, CAT, and GPx. These changes occurred—alongside reductions in SCORAD and DLQI scores [129]. In addition, significant reductions in SCORAD and DLQI scores were also observed from clinical studies, offering further insights into symptom relief, quality of life, and functional improvements that are essential for evaluating therapeutic efficacy in humans [142,143].

Subgroup analysis of clinical studies revealed that different types of antioxidant-rich whole foods or supplements have distinct effects on skin health outcomes. The long-chain structure of fatty acids, particularly omega-3 fatty acids, allows them to integrate into the phospholipid bilayer of cell membranes. This integration may enhance skin hydration and alleviate inflammatory skin conditions such as psoriasis by modulating eicosanoid synthesis [38,95,114,115]. Meanwhile, carotenoids are lipophilic molecules possessing a polyene chain structure, which enables them to quench singlet oxygen and provide photoprotection. In clinical studies, their utilization was linked to increased skin density, which may reflect improved dermal matrix integrity and collagen support [69,84,144]. Supplementation of vitamin D, which is a secosteroid hormone, was particularly effective in improving AD symptoms. This efficacy aligns with its known immunomodulatory role in skin homeostasis [121,123]. Collectively, these findings underscore the potential of condition-specific antioxidant strategies rather than a uniform intervention approach. 

Subgroup analyses revealed a divergence in the time required for significant cutaneous improvements between model types. Specifically, preclinical studies showed robust improvements in skin barriers and inflammatory markers within <12 weeks, whereas clinical outcomes required ≥12 weeks to achieve measurable structural benefits. In preclinical studies, interventions shorter than 12 weeks were sufficient to elicit significant changes in skin barrier function and inflammation status. Previous studies found that skin epidermal turnover time for hairless mice was 3.5 to 5 days on different body sites, and 8 to 10 days for normal mice [145,146,147]. This suggests that the skin responds relatively fast to antioxidants modulation due to faster metabolism and skin turnover, particularly under controlled experimental conditions and diet in animal models. In addition, animal studies often use higher doses than what is acceptable for humans. It also raises the possibility that short-term antioxidant interventions could be strategically applied during periods of heightened oxidative stress—such as during UV exposure or flare-ups of inflammatory skin conditions [30,31,148]. In contrast, clinical studies, interventions lasting 12 weeks or longer, resulted in significant and consistent improvements across multiple skin parameters, suggesting that sustained intake is necessary for measurable clinical benefits. This aligns with biological timelines for cutaneous remodeling. Firstly, healthy adults require 28 to 40 days for epidermal renewal, while aged skin may take 45 to 60 days. A 12-week period ensures completion of two to three full keratinization cycles, allowing for structural and functional improvements [9,149]. In addition, peak synthesis of collagen or elastin remodeling occurs at 8 to 12 weeks post-intervention, as demonstrated by retinoid studies showing new collagen deposition and elastic fiber reorganization [9]. Lastly, systemic antioxidants require 4 to 8 weeks to saturate skin tissues and neutralize oxidative stress in skin cells (e.g., ROS, lipid peroxidation), followed by gene modulation such as upregulation of collagen [150].

There was also a contrasting effect of antioxidants on epidermal thickness between preclinical and clinical studies. Both animal models and human skin exhibited epidermal thickening in response to ultraviolet B (UVB) exposure. This variation can be explained by species-specific differences in skin structure, photobiology, and experimental context. In preclinical studies, UVB irradiation is typically administered at high doses over several weeks prior to and during antioxidant intervention [58,59]. This repeated, high-intensity exposure induces marked epidermal hyperplasia, inflammation, and oxidative stress, thereby creating a model of acute photodamage [151]. Antioxidants in this setting reduce epidermal hyperplasia by attenuating inflammation and keratinocyte over proliferation, suggesting a protective effect [58,59]. In contrast, clinical studies generally use lower UVB doses, often administered only before and after the intervention period, and typically target sun-protected areas such as the back, which receive minimal cumulative UV exposure [33,144]. As a result, the skin response is milder, and antioxidants are more likely to support healthy epidermal regeneration and barrier repair. This may lead to a modest increase or maintenance of epidermal thickness, which is considered beneficial in the context of skin aging and overall skin health [33,76,144].

This systematic review and meta-analysis offer a comprehensive evaluation of the effects of antioxidant-rich whole foods and supplements on various skin disorders. A major strength of this study lies in its inclusion of both preclinical and clinical studies, enabling a more translational understanding of antioxidant mechanisms. By integrating both phenotypic and genotypic outcomes, the analysis captures both observable skin improvements and underlying molecular pathways related to inflammation and oxidative stress. Unlike previous reviews that focused solely on antioxidant supplements, this study takes a holistic approach by evaluating both dietary sources and supplements, thereby reflecting more realistic exposure patterns and potential dietary interventions.

Despite its comprehensive scope, this study has several limitations. Although the search strategy explicitly targeted both antioxidant-rich whole foods and supplements, the majority of eligible studies—particularly high-quality clinical trials—utilized supplements or concentrated extracts. Consequently, while our findings demonstrated the efficacy of antioxidant bioactives, the evidence specifically supporting “whole food” interventions remains less robust. This imbalance suggests that the current literature is heavily weighted towards standardized, high-dose delivery systems, which may not perfectly reflect the complex nutrient interactions and lower concentrations found in typical dietary patterns. Moreover, the inclusion of crossover and multi-arm trials may have influenced the precision of estimates. Although sensitivity analyses indicated that these designs did not materially alter the direction or significance of the findings, the potential for underestimated uncertainty is a limitation of the current synthesis. Future research with the application of whole foods will further delineate the effect of antioxidant-containing foods on skin health. Moreover, our bias-aware sensitivity analysis indicated that some outcomes, such as hydration, were sensitive to these small-study effects, further underscoring the need for large-scale, well-controlled dietary trials to strengthen the evidence base for public health recommendations. The discovery of distinct temporal patterns—where epidermal barrier repair occurs within weeks while dermal structural remodeling requires months—suggests the need for phase- and dose-specific strategies. For example, future protocols can apply utilization of higher-dose short-term loadings for acute inflammatory flare-ups, followed by lower-dose sustained whole-food intake for long-term anti-aging and dermal integrity. Furthermore, while current literature typically focuses on short-term interventions, there is a profound need for longitudinal studies that can explore the ‘synergistic effect’ of whole-food matrices over years rather than weeks. Such research would capture the cumulative benefits of complex nutrient interactions that are often missed in relatively short-term clinical trials.

## 5. Conclusions

This systematic review and meta-analysis provide comprehensive evidence that antioxidant interventions, primarily administered as dietary supplements, are associated with significant improvements in skin health, including enhanced barrier function, reduced oxidative stress, and mitigated inflammation. The role of whole food interventions appears promising but requires further investigation due to the limited number of studies. Additionally, variability in effects across antioxidant types and intervention duration highlights the need for personalized approaches.

## Figures and Tables

**Table 1:** PICOS criteria for inclusion of studies.

Parameter	Description
Population	Animals, Human subjects (>24 months);
Intervention	Antioxidant-rich whole food/supplement
Comparison	Placebo/not taking antioxidant-rich whole food/supplement
Outcomes	Primary: Skin health-related outcomes
	Secondary: Oxidative stress and inflammatory biomarkers, antioxidant capacity and antioxidant levels in blood
Study Design	Animal studies, Randomized controlled trials

**Table 2:** Effect of antioxidant-rich whole foods or supplements on preclinical skin health.

Outcome	Hedges’ g	95% CI
Hydration	1.75	[1.31; 2.20]
TEWL	−2.15	[−3.17; −1.13]
Epidermal thickness	−2.59	[−3.28; −1.89]
Hyaluronic acid	2.05	[0.93; 3.17]
Wrinkle formation	−4.28	[−5.79; −2.76]
Dermatitis score	−2.55	[−4.43; −0.67]
Pruritus score	−0.15	[−0.62; 0.32]

TEWL: Trans-Epidermal Water Loss.

**Table 3:** Effect of antioxidant-rich whole foods or supplements on clinical skin health.

Outcome	MD	95%CI
Hydration	2.12	[1.02; 3.21]
TEWL	−0.68	[−1.21; −0.16]
Epidermal thickness	0.12	[0.05; 0.19]
Skin density	0.68	[0.32; 1.04]
MED	21.56	[0.07; 43.04]
Sebum	−4.59	[−14.05; 4.88]
Skin elasticity	0.03	[0.00; 0.05]
SCORAD	−15.16	[−29.35; −0.97]
DLQI	−2.60	[−4.98; −0.23]
PASI	0.34	[−0.78; 1.46]
EASI	0.85	[−4.61; 6.30]

TEWL: trans-epidermal water loss; MED: minimal erythema dose; SCORAD: SCORing Atopic Dermatitis; DLQI: Dermatology Life Quality Index; PASI: psoriasis area and severity index; EASI: Eczema Area Severity Index.

## Data Availability

The data presented in this study will be made available on request from the corresponding author due to privacy concerns.

## Supplementary Materials

The following supporting information can be downloaded at: https://www.mdpi.com/article/10.3390/antiox15030301/s1, Table S1: Search terms and filters applied across different databases; Table S2: Study characteristics of preclinical studies; Table S3: Study characteristics of clinical studies with reports on skin aging; Table S4: Study characteristics of clinical studies with reports on inflammation skin condition; Table S5 Summary and classification of the antioxidant-rich whole foods or supplement type; Table S6: Effect of antioxidant-rich whole foods or supplements on preclinical skin health related biomarkers; Table S7: Sensitivity test of hydration in preclinical studies; Table S8: Sensitivity test of TEWL in preclinical studies; Table S9: Sensitivity test of Dermatitis score in preclinical studies; Table S10: Sensitivity test of Hyaluronic acid in preclinical studies; Table S11: Sensitivity test of IgE level in preclinical studies; Table S12: Sensitivity test of Epidermal thickness in preclinical studies; Table S13: Sensitivity test of Wrinkle formation in preclinical studies; Table S14: Sensitivity test of Pruritus score in preclinical studies; Table S15: Sensitivity test of SOD in preclinical studies; Table S16: Sensitivity test of CAT in preclinical studies; Table S17: Sensitivity test of GPx in preclinical studies; Table S18: Sensitivity test of IL-1β in preclinical studies; Table S19: Sensitivity test of IL-6 in preclinical studies; Table S20: Sensitivity test of TNF-α in preclinical studies; Table S21: Sensitivity test of hydration in clinical studies; Table S22: Sensitivity test of TEWL in clinical studies; Table S23: Sensitivity test of Epidermal thickness in clinical studies; Table S24: Sensitivity test of Skin density in clinical studies; Table S25: Sensitivity test of Skin sebum in clinical studies; Table S26: Sensitivity test of Skin elasticity in clinical studies; Table S27: Sensitivity test of SCORAD in clinical studies; Table S28: Sensitivity test of EASI in clinical studies; Table S29: Sensitivity test of DLQI in clinical studies; Table S30: Sensitivity test of PASI in clinical studies; Table S31: Sensitivity test of MED in clinical studies; Table S32: Bias-aware (Trim and Fill) sensitivity test for hydration in preclinical studies; Table S33: Bias-aware (Trim and Fill) sensitivity test for TEWL in preclinical studies; Table S34: Bias-aware (Trim and Fill) sensitivity test for epidermal thickness in preclinical studies; Table S35: Bias-aware (Trim and Fill) sensitivity test for dermatitis score in preclinical studies; Table S36: Bias-aware (Trim and Fill) sensitivity test for SOD in preclinical studies; Table S37: Bias-aware (Trim and Fill) sensitivity test for CAT in preclinical studies; Table S38: Bias-aware (Trim and Fill) sensitivity test for hydration in clinical studies; Table S39: Bias-aware (Trim and Fill) sensitivity test for TEWL in clinical studies; Table S40: Bias-aware (Trim and Fill) sensitivity test for skin elasticity in clinical studies; Table S41: Bias-aware (Trim and Fill) sensitivity test for DLQI in clinical studies; Table S42. Measurement Units and Statistical Models for Skin Outcomes in Clinical Studies; Figure S1: Forest plots of meta-analysis evaluating the effects of antioxidant-rich whole foods or supplements (AOX) consumption on animal skin health, with subgroup analysis based on duration of study cohorts. The effect of AOX consumption on (A) Skin hydration in preclinical studies; (B) Trans-epidermal water loss in preclinical studies; (C) Epidermal thickness in preclinical studies; (D) Hyaluronic acid in preclinical studies; (E) Wrinkle formation in preclinical studies; (F) IL-1 β mRNA level in preclinical studies; (G) IL-6 mRNA level in preclinical studies; (H) TNF-α mRNA level in preclinical studies; Figure S2: Forest plots of meta-analysis evaluating the effects of antioxidant-rich whole foods or supplement (AOX) consumption on human skin health, with subgroup analysis based on duration of study cohorts. The effect of AOX consumption on (A) Skin hydration in clinical studies; (B) Trans-epidermal water loss in clinical studies; (C) Skin elasticity in clinical studies; (D) SCORAD in clinical studies; (E) Epidermal thickness in clinical studies; (F) Skin density in clinical studies; (G) DLQI in clinical studies; Figure S3: Risk of bias assessment of preclinical studies; Figure S4: Risk of bias assessment of clinical studies (A) Parallel study; (B) Crossover study; Figure S5: Preclinical studies, Funnel plot of (A) Hydration (Egger’s test *p* < 0.0001); (B) Trans-epidermal water loss (Egger’s test *p* = 0.5923); (C) Epidermal thickness (Egger’s test *p* = 0.0012); (D) Dermatitis score (Egger’s test *p* = 0.0616); (E) SOD (Egger’s test *p* = 0.0009); (F) CAT (Egger’s test *p* = 0.0024); Figure S6: Clinical studies, Funnel plot of (A) Hydration (Egger’s test *p* = 0.0261); (B) Trans-epidermal water loss (Egger’s test *p* = 0.0216); (C) Epidermal thickness (Egger’s test *p* = 0.1397); (D) Skin elasticity (Egger’s test *p* = 0.4338); (E) DLQI (Egger’s test *p* = 0.1172).
