# Supplementary material for: Impact of Antioxidant-Rich Whole Foods or Supplements on Skin Health: A Systematic Review and Meta-Analysis of Preclinical and Clinical Studies
Source: Antioxidants (Basel). 2026 Feb 27;15(3):301. doi: 10.3390/antiox15030301 (PMC13024200; doi:10.3390/antiox15030301)
Supplement: Supplementary file 1 [file antioxidants-15-00301-s001.zip › antioxidants-4138642-supplementary.pdf]

Table S1. Search terms and filters applied across different databases

Database	Search terms	Filter
Pubmed	((((((((((((((((((((((antioxidants[Title/Abstract]) OR (dietary antioxidants[Title/Abstract])) OR (antioxidant supplementation[Title/Abstract])) OR (ascorbic acid[Title/Abstract])) OR (vitamin c[Title/Abstract])) OR (tocopherols[Title/Abstract])) OR (vitamin e[Title/Abstract])) OR (carotenoids[Title/Abstract])) OR (omega-3[Title/Abstract])) OR (vegetables[Title/Abstract])) OR (fruits[Title/Abstract])) OR (legumes[Title/Abstract])) AND (skin health[Title/Abstract])) OR (atopic dermatitides[MeSH Terms])) OR (atopic dermatitis[MeSH Terms])) OR (atopic eczema[MeSH Terms])) OR (acne[MeSH Terms])) OR (arthritic psoriasis[MeSH Terms])) OR (acne rosacea[MeSH Terms])) OR (skin aging[MeSH Terms])) OR (SKIN HYDRATION[Title/Abstract])) OR (SKIN ELASTICITY[Title/Abstract])) OR (Transepidermal water loss (TEWL[Title/Abstract]))) OR (skin firmness[Title/Abstract])) OR (skin texture[Title/Abstract]) OR (SCORAD[Title/Abstract]) OR (DLQI[Title/Abstract])	Clinical Trial, Randomized Controlled Trial, Humans, Other Animals, English
Embase	('antioxidant':ab,ti,kw OR 'dietary antioxidant index':kw,ti,ab OR 'antioxidant supplement':ab,ti,kw OR 'ascorbic acid':ab,kw,ti OR 'tocopherol':ab,kw,ti OR 'vitamin':ab,kw,ti OR 'carotenoid':ab,ti OR 'vegetable':ab,kw,ti OR 'fruits':ab,kw,ti OR 'legume':ab,kw,ti) AND ('skin health':ab,kw,ti OR 'atopic dermatitis':ab,kw,ti OR 'acne':ab,kw,ti OR 'psoriatic arthritis':ab,kw,ti OR 'rosacea':ab,kw,ti OR 'cutaneous parameters':ab,kw,ti OR 'skin hydration':ab,kw,ti OR 'skin elasticity':ab,kw,ti OR 'skin water loss':ab,kw,ti OR 'skin firmness'/exp OR 'skin firmness' OR 'skin texture':ab,kw,ti OR 'severity scoring of atopic dermatitis':ab,kw,ti OR 'dermatology life quality index':ab,kw,ti)	Embase (Not Medline)<br><br>Human<br><br>Nonhuman<br><br>Article
COCHRANE	(antioxidants OR dietary antioxidant OR antioxidant supplementation OR ascorbic acid or vitamin C OR tocopherol OR vitamin E OR carotenoids OR omega 3 OR legumes OR fruits or vegetables):ti,ab,kw)	In trials

	AND ((MeSH “Skin” OR MeSH “Dermatitis, Atopic” OR MeSH “Acne Vulgaris” OR MeSH “Arthritis, Psoriatic” OR MeSH “Rosacea” OR MeSH “Skin Aging” OR (skin hydration OR skin elasticity OR transepidermal water loss OR skin firmness OR skin texture OR SCORAD OR DLQI):ti,ab,kw	
CINAHL	(antioxidants OR dietary antioxidant OR antioxidant supplementation OR ascorbic acid or vitamin c OR tocopherol OR vitamin e OR carotenoids OR omega 3 OR legumes OR fruits or vegetables) AND AB (skin health OR atopic dermatitis OR atopic eczema OR acne OR arthritic psoriasis OR acne rosacea OR skin aging OR skin hydration OR skin elasticity OR transepidermal water loss OR skin firmness OR skin texture OR SCORAD OR DLQI)	English<br>Exclude Medline
Web of Science	(((TI=(("antioxidants") OR ("dietary antioxidants") OR ("antioxidant supplementation") OR ("ascorbic acid") OR ("vitamin c") OR ("tocopherols") OR ("vitamin e") OR ("carotenoids") OR ("omega-3") OR ("vegetables") OR ("fruits") OR ("legumes")))<br>OR (AK=(("antioxidants") OR ("dietary antioxidants") OR ("antioxidant supplementation") OR ("ascorbic acid") OR ("vitamin c") OR ("tocopherols") OR ("vitamin e") OR ("carotenoids") OR ("omega-3") OR ("vegetables") OR ("fruits") OR ("legumes")))<br>OR (AB=(("antioxidants") OR ("dietary antioxidants") OR ("antioxidant supplementation") OR ("ascorbic acid") OR ("vitamin c") OR ("tocopherols") OR ("vitamin e") OR ("carotenoids") OR ("omega-3") OR ("vegetables") OR ("fruits") OR ("legumes"))))<br>AND<br>((TI=(("skin health") OR ("atopic dermatitides") OR ("atopic dermatitis") OR ("atopic eczema") OR ("acne") OR ("arthritis psoriasis") OR ("acne rosacea") OR ("skin aging") OR ("skin hydration") OR ("skin elasticity") OR ("transepidermal water loss (TEWL)") OR ("skin firmness") OR ("skin texture") OR ("SCORAD") OR ("DLQI")))<br>OR (AK=(("skin health") OR ("atopic dermatitides") OR ("atopic dermatitis") OR ("atopic eczema") OR ("acne") OR ("arthritis psoriasis") OR ("acne	Article;<br>English

	rosacea") OR ("skin aging") OR ("skin hydration") OR ("skin elasticity") OR ("transepidermal water loss (TEWL)") OR ("skin firmness") OR ("skin texture") OR ("SCORAD") OR ("DLQI")))<br>OR (AB=(("skin health") OR ("atopic dermatitides") OR ("atopic dermatitis") OR ("atopic eczema") OR ("acne") OR ("arthritis psoriasis") OR ("acne rosacea") OR ("skin aging") OR ("skin hydration") OR ("skin elasticity") OR ("transepidermal water loss (TEWL)") OR ("skin firmness") OR ("skin texture") OR ("SCORAD") OR ("DLQI")))))	

Table S2 Study characteristics of preclinical studies

Reference	Study design	Duration	Animal type	Subject size (M/F)	Intervention food description	Control food description	Health status	Main Result
Lee et al. (2024)	Parallel	8 weeks	SKH-1 hairless mice	16 (16/0) I: n=8; C: n=8	AIN93G supplemented 100 mg/kg BW of IB (Indian gooseberry extract powder: barley sprout juice powder=2:1)	Normal meal	Healthy with UVB exposure	Skin hydration; TEWL; Hyaluronic acid; Epidermal thickness; Wrinkle formation; SOD; CAT; GPx
Lee et al. (2024)	Parallel	8 weeks	SKH-1 hairless mice	16 (16/0) I: n=8; C: n=8	AIN93G supplemented 200 mg/kg BW of IB (Indian gooseberry extract powder: barley sprout juice powder=2:1)	Normal meal	Healthy with UVB exposure	Skin hydration; TEWL; Hyaluronic acid; Epidermal thickness; Wrinkle formation; SOD; CAT; GPx
Lee et al. (2024)	Parallel	8 weeks	SKH-1 hairless mice	16 (16/0) I: n=8; C: n=8	AIN93G supplemented 400 mg/kg BW of IB (Indian gooseberry extract powder: barley sprout juice powder=2:1)	Normal meal	Healthy with UVB exposure	Skin hydration; TEWL; Hyaluronic acid; Epidermal thickness; Wrinkle formation; SOD; CAT; GPx
Geng et al.	Parallel	14 weeks	SKH-1	16 (16/0)	AIN93G supplement	Normal meal	Healthy	Skin hydration;

(2024)			hairless mice	I: n=8; C: n=8	with 0.025% IE (Isoeugenol)		with UVB exposure	Hyaluronic acid; Epidermal thickness; Wrinkle formation
Park et al. (2024)	Parallel	12 weeks	SPF Hos:HR-1 hairless mice	16 (0/16) I: n=8; C: n=8	Canola meal extract, 250 mg/kg BW/day	Normal meal	Healthy with UVB exposure	Epidermal thickness; Wrinkle number; Total wrinkle length; SOD; CAT
Park et al. (2024)	Parallel	12 weeks	SPF Hos:HR-1 hairless mice	16 (0/16) I: n=8; C: n=8	Canola meal extract, 500 mg/kg BW/day	Normal meal	Healthy with UVB exposure	Epidermal thickness; Wrinkle number; Total wrinkle length; SOD; CAT
Park et al. (2024)	Parallel	12 weeks	SPF Hos:HR-1 hairless mice	16 (0/16) I: n=8; C: n=8	Canola meal extract, 1000 mg/kg BW/day	Normal meal	Healthy with UVB exposure	Epidermal thickness; Wrinkle number; Total wrinkle length; SOD; CAT
Im et al. (2019)	Parallel	12 weeks	Hos:HR-1 hairless mice	12 (12/0) I: n=6; C: n=6	Oxya chinensis sinuosa Mishchenko extracts	Normal meal	Healthy with UVB exposure	TEWL; Hyaluronic acid; Epidermal thickness; SOD; CAT; IL-1β mRNA; IL-6 mRNA; TNF-α mRNA
Lee et al. (2018)	Parallel	12 weeks	Hos:HR-1 hairless mice	14 (14/0) I: n=7; C: n=7	1000 mg/kg hesperidin	Normal meal	Healthy with UVB exposure	TEWL; Epidermal thickness
Kim et al. (2020)	Parallel	4 weeks	NC/Nga mice	16	250 mg/kg BW Fermented Morinda citrifolia (Noni)	Normal meal	DNCB induced atopic	Dermatitis score; Epidermal thickness; IL-6 mRNA; TNF-α mRNA;

							dermatitis	Serum IgE
Kim et al. (2020)	Parallel	4 weeks	NC/Nga mice	16	500 mg/kg BW Fermented Morinda citrifolia (Noni)	Normal meal	DNCB induced atopic dermatitis	Dermatitis score; Epidermal thickness; IL-6 mRNA; TNF-α mRNA; Serum IgE
Kim et al. (2020)	Parallel	4 weeks	NC/Nga mice	16	1000 mg/kg BW Fermented Morinda citrifolia (Noni)	Normal meal	DNCB induced atopic dermatitis	Dermatitis score; Epidermal thickness; IL-6 mRNA; TNF-α mRNA; Serum IgE
Kim et al. (2021)	Parallel	14 weeks	SPF Hos:HR-1 hairless mice	12 (0/12) I: n=6; C: n=6	50 mg/kg BW evening primrose sprout (Oenothera biennis l.) extract	Normal meal	Healthy with UVB exposure	TEWL; Epidermal thickness; Wrinkle formation; Wrinkle number; Total wrinkle length
Kim et al. (2021)	Parallel	14 weeks	SPF Hos:HR-1 hairless mice	12 (0/12) I: n=6; C: n=6	200 mg/kg BW evening primrose sprout (Oenothera biennis l.) extract	Normal meal	Healthy with UVB exposure	TEWL; Epidermal thickness; Wrinkle formation; Wrinkle number; Total wrinkle length
Kim & Choung (2012)	Parallel	4 weeks	NC/Nga mice	16	Polyphenols and anthocyanins (2.56: 7.31 ug/kg/BW)	Normal meal	DNCB induced atopic dermatitis	Dermatitis score; Serum IgE

Kim & Choung (2012)	Parallel	4 weeks	NC/Nga mice	16	Polyphenols and anthocyanins (5.12: 14.62 ug/kg/BW)	Normal meal	DNCB induced atopic dermatitis	Dermatitis score; Serum IgE
Kim & Choung (2012)	Parallel	4 weeks	NC/Nga mice	16	Polyphenols and anthocyanins (10.25: 29.25 ug/kg/BW)	Normal meal	DNCB induced atopic dermatitis	Dermatitis score; Serum IgE
Harauma et al. (2024)	Parallel	14 days	BALB/c mice	15 I: n=10; C: n=5	0.88 g/kg diet of Omega-3 fatty acid	Normal meal	Healthy with UVB exposure	Skin hydration; TEWL; Epidermal thickness
Harauma et al. (2024)	Parallel	14 days	BALB/c mice	14 I: n=9; C: n=5	0.12 g/kg diet of Omega-3 fatty acid	Normal meal	Healthy with UVB exposure	Skin hydration; TEWL; Epidermal thickness
Hiragun et al. (2016)	Parallel	8 weeks	HR-1 hairless mice	12 (12/0) I: n=6; C: n=6	34 mg zinc/100g diet and 0.14 mg magnesium/100g diet	Normal diet with zinc (67 mg/100g diet) and magnesium (0.25 mg/100g diet)	DNCB induced atopic dermatitis	Skin hydration; TEWL; Serum IgE
Hiragun et al. (2016)	Parallel	8 weeks	HR-1 hairless mice	12 (12/0) I: n=6; C: n=6	34 mg zinc/100g diet and 0.14 mg magnesium/100g diet + 1 mg β-carotene/100g diet	Normal diet with zinc (67 mg/100g diet) and magnesium (0.25 mg/100g diet)	DNCB induced atopic dermatitis	Skin hydration; TEWL; Serum IgE

Hiragun et al. (2016)	Parallel	8 weeks	HR-1 hairless mice	12 (12/0) I: n=6; C: n=6	34 mg zinc/100g diet and 0.14 mg magnesium/100g diet + 1 mg lycopene/100g diet	Normal diet with zinc (67 mg/100g diet) and magnesium (0.25 mg/100g diet)	DNCB induced atopic dermatitis	Skin hydration; TEWL; Serum IgE
Jeon et al. (2010)	Parallel	8 weeks	HWY/Slc hariless rats	10 (0/10) I: n=5; C: n=5	1500 ppm EGCG	Normal diet	Healthy with UVB exposure	TEWL; Epidermal thickness
Zhou et al. (2016)	Parallel	24 days	BALB/c mice	12 (0/12) I: n=6; C: n=6	10 mg/kg BW Esculeoside A	Normal diet	DNCB induced atopic dermatitis	Dermatitis score
Zhou et al. (2016)	Parallel	24 days	BALB/c mice	12 (0/12) I: n=6; C: n=6	50 mg/kg BW Esculeoside A	Normal diet	DNCB induced atopic dermatitis	Dermatitis score
Zhou et al. (2016)	Parallel	24 days	BALB/c mice	12 (0/12) I: n=6; C: n=6	100 mg/kg BW Esculeoside A	Normal diet	DNCB induced atopic dermatitis	Dermatitis score
Horie et al. (2024)	Parallel	8 weeks	TSOD mice	12 I: n=6; C: n=6	0.1 ml/10 g/day linseed oil	Normal diet	TSOD mice	TEWL; Epidermal thicknes
Viyoch et al. (2012)	Parallel	12 weeks	ICR mice	8 (8/0) I: n=4; C: n=4	0.5 mg M. sapientum fruit pulps/g bw/day	Normal diet	Healthy with UVB exposure	Epidermal thickness; Skin elasticity

Viyoch et al. (2012)	Parallel	12 weeks	ICR mice	8 (8/0) I: n=4; C: n=4	1.0 mg M. sapientum fruit pulps/g bw/day	Normal diet	Healthy with UVB exposure	Epidermal thickness; Skin elasticity
Viyoch et al. (2012)	Parallel	12 weeks	ICR mice	8 (8/0) I: n=4; C: n=4	1.5 mg M. sapientum fruit pulps/g bw/day	Normal diet	Healthy with UVB exposure	Epidermal thickness; Skin elasticity
Viyoch et al. (2012)	Parallel	12 weeks	ICR mice	8 (8/0) I: n=4; C: n=4	0.5 mg M. suerier's fruit pulps/g bw/day	Normal diet	Healthy with UVB exposure	Epidermal thickness; Skin elasticity
Viyoch et al. (2012)	Parallel	12 weeks	ICR mice	8 (8/0) I: n=4; C: n=4	1.0 mg M. suerier's fruit pulps/g bw/day	Normal diet	Healthy with UVB exposure	Epidermal thickness; Skin elasticity
Viyoch et al. (2012)	Parallel	12 weeks	ICR mice	8 (8/0) I: n=4; C: n=4	1.5 mg M. suerier's fruit pulps/g bw/day	Normal diet	Healthy with UVB exposure	Epidermal thickness; Skin elasticity
Park et al. (2023)	Parallel	8 weeks	SKH-1 hairless mice	16 (16/0) I: n=8; C: n=8	5 mg wheat ceramide powder/kg BW	Normal diet	Healthy with UVB exposure	Skin hydration; Epidermal thickness; Wrinkle formation; SOD; CAT; GPx: IL-1β mRNA IL-6 mRNA; TNF-α mRNA
Park et al. (2023)	Parallel	8 weeks	SKH-1 hairless mice	16 (16/0) I: n=8; C: n=8	20 mg wheat ceramide powder/kg BW	Normal diet	Healthy with UVB exposure	Skin hydration; Epidermal thickness; Wrinkle formation; SOD; CAT; GPx: IL-1β mRNA IL-6 mRNA; TNF-α

								mRNA
Park et al. (2023)	Parallel	8 weeks	SKH-1 hairless mice	16 (16/0) I: n=8; C: n=8	100 mg wheat ceramide powder/kg BW	Normal diet	Healthy with UVB exposure	Skin hydration; Epidermal thickness; Wrinkle formation; SOD; CAT; GPx: IL-1β mRNA IL-6 mRNA; TNF-α mRNA
Park et al. (2021)	Parallel	6 weeks	HRM-2 mice	12 (12/0) I: n=6; C: n=6	100 mg Zingiber mioga Extract/kg BW	Normal diet	Healthy with UVB exposure	Skin hydration; SOD; CAT; GPx; IL-1β mRNA IL-6 mRNA; TNF-α Mrna; Collagen type 1 mRNA
Park et al. (2021)	Parallel	6 weeks	HRM-2 mice	12 (12/0) I: n=6; C: n=6	200 mg Zingiber mioga Extract/kg BW	Normal diet	Healthy with UVB exposure	Skin hydration; SOD; CAT; GPx; IL-1β mRNA IL-6 mRNA; TNF-α Mrna; Collagen type 1 mRNA
Fujii et al. (2013)	Parallel	7 weeks	Hos: HR-1 hairless mice	16 (16/0) I: n=8; C: n=8	5% Amla extract of diet	Normal diet	Healthy with UVB exposure	Skin hydration; TEWL
Tamaru et al. (2020)	Parallel	7 weeks	Hos: HR-1 hairless mice	14 (14/0) I: n=7; C: n=7	200 mg immature Citrus unshiu powder/kg BW	Normal diet	Healthy with UVB exposure	Skin hydration; TEWL; Epidermal thickness

Noli et al. (2007)	Parallel	60 days	Dogs with perennial AD	9 (4/5) I: n=4; C: n=5	Conjugated linoleic acid and black currant seed oil 90 mg/kg BW	Placebo	Dogs with perennial AD	Dermatitis score; Skin pruritus score
Noli et al. (2007)	Parallel	60 days	Dogs with perennial AD	10 (4/6) I: n=5; C: n=5	Conjugated linoleic acid 90 mg/kg BW	Placebo	Dogs with perennial AD	Dermatitis score; Skin pruritus score
Noli et al. (2007)	Parallel	60 days	Dogs with perennial AD	11 (2/9) I: n=6; C: n=5	Black currant seed oil 90 mg/kg BW	Placebo	Dogs with perennial AD	Dermatitis score; Skin pruritus score
Bensignor et al. (2008)	Crossover	60 days	Dogs with perennial AD	16	1.6%E total n6 EFAs enriched diet	Placebo	Dogs with perennial AD	Dermatitis score; Skin pruritus score
Bensignor et al. (2008)	Crossover	60 days	Dogs with perennial AD	16	0.32%E total n3 EFAs enriched diet	Placebo	Dogs with perennial AD	Dermatitis score; Skin pruritus score
Harvey (1999)	Parallel	8 weeks	Dogs with perennial AD	13 I: n=6; C: n=7	176 mg/kg Borage oil + 22 mg/kg fish oil + 42 mg/kg GLA + 4.2 mg/kg EPA + 2.7 mg/kg/BW	204 mg/kg olive oil/BW	Dogs with perennial AD	Skin pruritus score
Harvey (1999)	Parallel	8 weeks	Dogs with perennial AD	13 I: n=6; C: n=7	88 mg/kg Borage oil + 11 mg/kg fish oil + 102 mg/kg olive oil + 21 mg/kg GLA + 2.1 mg/kg	204 mg/kg olive oil/BW	Dogs with perennial AD	Skin pruritus score

Mueller et al. (2004)	Parallel	10 weeks	Dogs with perennial AD	20<br>I: n=10;<br>C: n=10	327 mg/kg omega-3 + 468 mg/kg omeg-6/BW	84 mg/kg omega-3 + 509 mg/kg omeg-6/BW	Dogs with perennial AD	Dermatitis score
Mueller et al. (2004)	Parallel	10 weeks	Dogs with perennial AD	20<br>I: n=10;<br>C: n=10	141 mg/kg omega-3 + 426 mg/kg omeg-6/BW	84 mg/kg omega-3 + 509 mg/kg omeg-6/BW	Dogs with perennial AD	Dermatitis score
Kim et al. (2019)	Parallel	4 weeks	SKH-1 hairless mice	12 (0/12)<br>I: n=6;<br>C: n=6	158 mg/kg blackberry/ BW	Normal diet	Healthy with UVB exposure	Epidermal thickness
Kim et al. (2019)	Parallel	4 weeks	SKH-1 hairless mice	12 (0/12)<br>I: n=6;<br>C: n=6	158 mg/kg fermented blackberry/BW	Normal diet	Healthy with UVB exposure	Epidermal thickness
Song et al. (2013)	Parallel	12 weeks	HR-1 hairless mice	10<br>I: n=5;<br>C: n=5	100 mg/kg mango extracts/BW	Normal diet	Healthy with UVB exposure	Epidermal thickness
Ye et al. (2014)	Parallel	42 days	Kunming mice	20 (10/10)<br>I: n=10;<br>C: n=10	100 mg/kg Idesia polycarpa Defatted Fruit Residue extracts/BW	Normal diet	Aging mice	Skin hydration; SOD; CAT; GPx
Ye et al. (2014)	Parallel	42 days	Kunming mice	20 (10/10)<br>I: n=10;<br>C: n=10	200 mg/kg Idesia polycarpa Defatted Fruit Residue extracts/BW	Normal diet	Aging mice	Skin hydration; SOD; CAT; GPx

Ye et al. (2014)	Parallel	42 days	Kunming mice	20 (10/10) I: n=10; C: n=10	400 mg/kg Idesia polycarpa Defatted Fruit Residue extracts/BW	Normal diet	Aging mice	Skin hydration; SOD; CAT; GPx

DNCB: 2,4-dinitrochlorobenzene; **SPF:** Specific-Pathogen-Free; **NC/Nga mice:** Nihon Clea/Nihon Genetic Animal; **BALB/c:** Bagg Albino Laboratory-Bred Mice, substrain c; **ICR:** Institute of Cancer Research; **HRM-2:** Hairless Recombination Mutant 2 mice; **TSOD:** Tsumura Suzuki Obese Diabetes; **HWY/Slc:** Hairless Wistar Yagi rats; **TEWL:** Trans-Epidermal Water Loss; **SOD:** Superoxide dismutase; **CAT:** catalase; **GPx:** glutathione peroxidase; **IL-1β:** Interleukin-1 beta; **IL-6:** Interleukin-6; **TNF-α:** Tumor Necrosis Factor alpha; **IgE:** immunoglobulin E.

Table S3 Study characteristics of clinical studies with reports on skin aging

Reference	Study design	Duration	Subject number (M/F)	Intervention food description	Control food description	Health status	Main Result
De Sprit et al. (2012)	Parallel	12 weeks	52 (0/52) I: n=26; C: n=26	Supplements contain: 7.5 mg β-carotene, 46 mg vitamin E, 200 mg vitamin C, 0.4 mg folic acid	Placebo	Healthy female	Skin hydration; TEWL; Epidermal thickness; Skin density; Serum lutein; Serum zeaxanthin; Serum cryptoxanthin; Serum lycopene; Serum α-carotene; serum β-carotene; Serum α-Tocopherol; Serum Retinol
Jenkins et al. (2014)	Parallel	14 weeks	106 (0/106) I: n=51; C: n=55	Test meal A contain: 70 mg isoflavone, 8 mg lycopene, 250 mg vitamin C, 250 mg vitamin E; 660 mg Omega-3 EFA	Placebo	Healthy postmenopausal female	Skin hydration; TEWL; Serum lycopene; Serum α-carotene; Serum vitamin C; Serum EPA; Serum DHA
Jenkins et al. (2014)	Parallel	14 weeks	108 (0/108) I: n=53; C: n=55	Test meal B contain: 40 mg isoflavone, 3 mg lycopene, 180 mg vitamin C, 30 mg vitamin E; 660 mg Omega-3 EFA	Placebo	Healthy postmenopausal female	Skin hydration; TEWL; Serum lycopene; Serum α-carotene; Serum vitamin C; Serum EPA; Serum DHA
Heinrich et al. (2006)	Parallel	12 weeks	26 (0/26) I: n=13; C: n=13	Supplements A contain: 3 mg lycopene, 3 mg lutein, 4.8 mg β-carotene, 10 mg α-tocopherol, 0.075 mg selenium	Placebo	Healthy female	Epidermal thickness; Skin density; Serum lutein; Serum zeaxanthin; Serum lycopene; Serum β-carotene; Serum α-

							Tocopherol
Heinrich et al. (2006)	Parallel	12 weeks	26 (0/26) I: n=13; C: n=13	Supplements B contain: 6 mg lycopene, 4.8 mg β-carotene, 10 mg α-tocopherol, 0.075 mg selenium	Placebo	Healthy female	Epidermal thickness; Skin density; Serum lutein; Serum zeaxanthin; Serum lycopene; Serum β-carotene; Serum α-Tocopherol
Segger et al. (2008)	Parallel	12 weeks	24 (0/24) I: n=12; C: n=12	10 mL (9.2g) test oil: fish oil Eskimo-3 (70%), evening primrose oil (20%), canola oil (10%), vitamin D 40 IU/ml. EPA (12.7%), DHA (8.1%), GLA (2.1%)	Placebo	Healthy female	TEWL; Skin elasticity
Segger & Schönlau (2004)	Parallel	12 weeks	58 (0/58) I: n=29; C: n=29	Supplement: Pycnogenol (10 mg), vitamin C (30 mg), vitamin E (d-a-tocopherylacetate)(5 mg), biotin (75 mg), selenium (25 mg), zinc as gluconate (7.5 mg), bio-marine complex (hydrolised collagen and glycosaminoglycans from salmon) (50 mg), horsetail herb extract (natural source of silicate) (40 mg), blueberry extract (15 mg) and tomato extract (dietary carotenoids, b and c-carotene, lycopene and	Placebo	Healthy female	Skin elasticity

				lutein) (34 mg).			
Yoon et al. (2014)	Parallel	12 weeks	44 (0/44) I: n=22; C: n=22	2 mg Astaxanthin	Placebo	Healthy female	Skin hydration; TEWL
Handeland et al. (2024)	Parallel	12 weeks	51 I: n=26; C: n=25	1g krill oil (170mg EPA, 90mg DHA)	Placebo	Healthy female	Skin hydration; TEWL; Skin elasticity
Handeland et al. (2024)	Parallel	12 weeks	54 I: n=29; C: n=25	2g krill oil (340mg EPA, 180mg DHA)	Placebo	Healthy female	Skin hydration; TEWL; Skin elasticity
Ito et al. (2018)	Parallel	9 weeks	22 (2/20) I: n=11; C: n=11	4 mg Astaxanthin	Placebo	Healthy adults	Skin hydration; TEWL
Choi et al. (2018)	Parallel	12 weeks	67 I: n=39; C: n=38	400 mg fermented Cyclopia intermedia extract	Placebo	Healthy adults	Skin hydration; TEWL; Wrinkle formation; Skin elasticity
Choi et al. (2018)	Parallel	12 weeks	66 I: n=38; C: n=38	800 mg fermented Cyclopia intermedia extract	Placebo	Healthy adults	Skin hydration; TEWL; Wrinkle formation; Skin elasticity
Foolad et al. (2019)	Parallel	16 weeks	31 (0/31) I: n=16; C: n=15	20%E almond consumption (2.1 oz/58.9g)	Nut-free snack	Postmeno pausal females	TEWL; Sebum; Wrinkle formation
Xie et al. (2022)	Parallel	12 weeks	55 I: n=29; C: n=26	70 mg Cucumis melo extract, 60 mg Acerola extract, 10 mg Lyophillized olive fruit, 20 mg Lyophillized aloe vera gel, 5 mg	Placebo	Healthy adults	Skin hydration; TEWL; Sebum; Skin pH; Skin elasticity

				Grape seed extract, 0.5 mg Lycopene			
Cho et al. (2010)	Parallel	90 days	29 (0/29) I: n=14; C: n=15	90 mg β-carotene	30 mg β-carotene	Healthy female	Skin elasticity; Skin MED
Puch et al. (2008)	Parallel	6 months	72 (0/72) I: n=36; C: n=36	300 mg Gamma-linolenic acid, 47 mg catechins, 2 mg Vitamin E	Placebo	Healthy female	TEWL; Serum LA
Tseng et al. (2022)	Parallel	8 weeks	40 I: n=20; C: n=20	730 ml coffee pulp drink	Placebo	Healthy adults	Skin hydration; Wrinkle formation
Tarshish et al. (2023)	Parallel	12 weeks	59 (0/59) I: n=30; C: n=29	10 mg carotenoids from Lumenato oral supplement	Placebo	Healthy female	TEWL
Bertuccelli et al. (2016)	Parallel	90 days	60 (0/60) I: n=30; C: n=30	Antioxidant cocktail: 10 mg trans-resveratrol, 60 µg selenium, 10 mg vitamin E and 50 mg vitamin C	Placebo	Healthy female	Skin hydration; Skin elasticity; SOD; MDA
Heinrich et al. (2011)	Parallel	12 weeks	60 (0/60) I: n=30; C: n=30	1402 total catechin: 100 mg epicatechin, 23.2 mg catechin, 980 mg EGCG	Placebo	Healthy female	Skin hydration; TEWL; Epidermal thickness; Skin density; Wrinkle formation; Serum epicatechin; Serum catechin; serum EGCG
Maruki-Uchida et al. (2018)	Parallel	8 weeks	32 (0/32) I: n=16; C: n=16	5 mg piceatannol from Passion Fruit Seed Extract	Placebo	Healthy female	Skin hydration; TEWL; Skin elasticity

Zhao et al. (2021)	Crossover	12 weeks	76	100 mg Pycnogenol@ extract from the bark of the French maritime pine	Placebo	Healthy adults	Skin hydration; TEWL; Skin elasticity
Heinrich et al. (2006)	Parallel	12 weeks	24 (0/24) I: n=12; C: n=12	328.5 mg Flavanol cocoa: 61.1 mg epicatechin + 20.4 mg catechin	Placebo	Healthy female	Skin hydration; TEWL; Epidermal thickness; Wrinkle formation
Chiu et al. (2016)	Crossover	6 months	42 (17/25)	9.1959g total phenolics: 6419 mg flavonoids	Placebo	Healthy adults	Skin hydration; TEWL; Wrinkle formation; Skin elasticity; CAT; GPx
Mogollon et al. (2014)	Parallel	12 weeks	74 (0/74) I: n=33; C: n=41	600 mg flavanols	Placebo	Healthy female	Skin hydration; Skin elasticity
Tumsutti et al. (2022)	Parallel	12 weeks	101 (0/101) I: n=50; C: n=51	100 mg Glycine max (soya bean)	Placebo	Healthy postmenopausal female	Skin hydration; TEWL; Skin elasticity; MDA
Chakkalakal et al. (2023)	Parallel	8 weeks	38 (0/38) I: n=21; C: n=17	500 mg Terminalia chebula Fruit Extracts	Placebo	Healthy adults	Sebum; Wrinkle formation
Sato et al. (2023)	Parallel	12 weeks	213 (101/112) I: n=107; C: n=106	2200 mg DDMP (2,3-dihydro-3,5-dihydroxy-6-methyl-4H-pyran-4-one)	Placebo	Healthy adults	Skin hydration; TEWL
Kappler et al. (2022)	Parallel	56 days	60 (0/60) I: n=30; C: n=30	175 mg Blue Fenugreek Kale Extract	Placebo	Healthy female	Skin hydration; TEWL

Nobile et al. (2021)	Parallel	12 weeks	100 (0/100) I:n=50; C: n=50	250 mg polyphenolic extracts	Placebo	Healthy female	Skin hydration; TEWL; Sebum; Skin elasticity; MDA
Tominaga et al. (2017)	Parallel	16 weeks	43 (0/43) I: n=22; C: n=21	6 mg astaxanthin	Placebo	Healthy female	Skin elasticity
Tominaga et al. (2017)	Parallel	16 weeks	43 (0/43) I: n=22; C: n=21	12 mg astaxanthin	Placebo	Healthy female	Skin elasticity
Yoon et al. (2016)	Parallel	24 weeks	62 (0/62) I: n=31; C: n-31	320 mg cocoa flavanols	Placebo	Healthy female	Skin hydration; TEWL; Skin elasticity; MED
Chakkalakal et al. (2022)	Parallel	4 weeks	18 (3/15) I: n=8; C: n=10	75 mg punicalagin	Placebo	Healthy adults	TEWL; Sebum
Rybak et al. (2021)	Parallel	24 weeks	56 (0/56) I: n=28; C: n=28	20%E Almond consumption	Placebo	Healthy postmenop ausal female	Skin hydration; TEWL; Sebum
De Spirt et al. (2009)	Parallel	12 weeks	30 (0/30) I: n=15; C: n=15	2.2 g flaxseed oil: 1161.6 mg ALA, 431.2 mg oleic acid, 352 mg LA	Placebo	Healthy female	Skin hydration; TEWL; Wrinkle formation
De Spirt et al. (2009)	Parallel	12 weeks	30 (0/30) I: n=15; C: n=15	2.2 g borage oil: 8.36 mg ALA, 330 mg oleic acid, 851.4 mg LA, 475.2 mg GLA	Placebo	Healthy female	Skin hydration; TEWL; Wrinkle formation
Kim et al.	Parallel	12 weeks	92 (0/92)	600 mg Boesenbergia pandurata	Placebo	Healthy	Skin hydration; Skin

(2017)			I: n=46; C: n=46	extract		adults	elasticity
Žmitek et al. (2017)	Parallel	12 weeks	22 (0/22) I: n=11 C: n=11	50 mg Coenzyme Q10	Placebo	Healthy female	Skin hydration; Wrinkle formation; MED
Žmitek et al. (2017)	Parallel	12 weeks	21 (0/21) I: n=10 C: n=11	150 mg Coenzyme Q10	Placebo	Healthy female	Skin hydration; Wrinkle formation; MED
Uchiyama et al. (2019)	Parallel	12 weeks	64 (0/64) I: n=31 C: n=33	25 mg dry lingoberry extract + 30 mg dry amla fruit extract	Placebo	Healthy female	Skin hydration; TEWL; Epidermal thickness
Nobile et al. (2024)	Parallel	84 days	66 (0/66) I: n=33 C: n=33	4 mg polyphenols from 100 mg fermented bilberry extract	Placebo	Healthy female	Skin elasticity
Ham et al. (2022)	Parallel	12 weeks	80 (0/80) I: n=40; C: n=40	300 mg Green Madarin (putgyul) extract	Placebo	Healthy females	Skin hydration; TEWL
Rizzo et al. (2023)	Parallel	24 weeks	44 (0/44) I: n=23; C: n=21	50 mg isoflavones	Placebo	Healthy postmenop ausal female	Skin hydration; Sebum
Hoshino et al. (2019)	Parallel	8 weeks	60 (15/45) I: n=30; C: n=30	180 mg (catechin-equivalent) of proanthocyanidins derived from Acacia bark	Placebo	Healthy adults	Skin hydration; TEWL
Li et al. (2021)	Parallel	12 weeks	29 (0/29) I: n=13;	42.5243 g Almond consumption	Placebo	Healthy female	Skin hydration; Sebum; MED

			C: n=16				
Sanjaya et al. (2024)	Parallel	12 weeks	29 (14/15) I: n=14; C: n=15	2 mg Wine Lees Extract -derived ceramides and glucosylceramides	Placebo	Healthy adults	Skin hydration; TEWL
Thom et al. (2005)	Parallel	6 months	40 (0/40) I: n=20; C: n=20	712 mg supplements: 350 marine protein complex + 100 a-lipoic acid + vitamin c + 62 red clover extract (8%) + 40 tomato extract (5% lycopene) + 30 pine bark extract (95%) + 18 vitamin E + 18 vitamin B3 + 12 soya extract (40%) + 12 zinc + 8 vitamin B5 + 2 copper	Placebo	Healthy female	Epidermal thickness
Nobile et al. (2022)	Parallel	56 days	110 (44/66) I: n=55 C: n=55	410 mg red orange Complex	Placebo	Healthy adults	Skin hydration; TEWL; Wrinkle formation; Skin elasticity; MED; MDA
Lopresti et al. (2024)	Parallel	12 weeks	100 (21/79) I: n=50; C: n=50	271 mg supplements: 9 mg astaxanthin, 250 mg grape juice extract, and 12 mg vitamin E (d-alpha tocopherol)	Placebo	Healthy adults	Skin carotenoids; MDA; TNF-α mRNA; IL-6 mRNA
Tanaka et al. (2016)	Parallel	12 weeks	64 (0/64) I: n=32; C: n=32	0.04 mg aloe sterol/100g yogurt	Placebo	Healthy female	Skin hydration; TEWL; Skin elasticity
Bouilly-Gauthier et al. (2010)	Parallel	6 weeks	43	7.2 mg carotenoids	Placebo	Healthy female	MED

Myung et al. (2020)	Parallel	12 weeks	101<br>I: n=50<br>C: n=51	300 mg Hydrangea serrata (Thunb.) Ser. Leaves Extract	Placebo	Healthy adults	Skin hydration; Skin elasticity
Myung et al. (2020)	Parallel	12 weeks	101<br>I: n=50<br>C: n=51	600 mg Hydrangea serrata (Thunb.) Ser. Leaves Extract	Placebo	Healthy adults	Skin hydration; Skin elasticity
Skaug et al. (2014)	Parallel	6 weeks	37<br>I: n=18;<br>C: n=19	1940.206 mg multivitamins: 1.25 VA, 110 VE, 420 VC, 29.8 VB, 500 zinc	Placebo	Athletes	Skin carotenoids
Rizwan et al. (2011)	Parallel	12 weeks	17<br>I: n=9;<br>C: n=8	55 g tomato paste + 10 g olive oil	Placebo	Healthy female	MED
Baswan et al. (2020)	Parallel	12 weeks	60<br>I: n=31;<br>C: n=29	19.569 mg multi-carotenoids/day: 4.25 β-carotene, 1.1 α-carotene, 1.12 lutein, 0.053 zeaxanthin	Placebo	Healthy adults	MED

TEWL: Trans-Epidermal Water Loss; **SOD:** Superoxide dismutase; **CAT:** catalase; **GPx:** glutathione peroxidase; **IL-1β:** Interleukin-1 beta; **IL-6:** Interleukin-6; **TNF-α:** Tumor Necrosis Factor alpha; **IgE:** immunoglobulin E; **SCORAD:** SCORing Atopic Dermatitis; **DLQI:** Dermatology Life Quality Index; **PASI:** psoriasis area and severity index; **EASI:** Eczema Area Severity Index; **MDA:** Malondialdehyde; **MED:** Minimal Erythema Dose; **EPA:** Eicosapentaenoic Acid; **DHA:** Docosahexaenoic Acid; **LA:** Linoleic Acid; **EGCG:** Epigallocatechin Gallate; **BSA:** Body Surface Activity; **DAS28-CRP:** Disease Activity Score 28 - C-Reactive Protein; **TAC:** total antioxidant capacity; **25(OH)D:** 25-Hydroxyvitamin D.

Table S4 Study characteristics of clinical studies with reports on inflammation skin condition

Reference	Study design	Duration	Subject number (M/F)	Intervention food description	Control food description	Health status	Main Result
Leite et al. (2022)	Parallel	12 weeks	65 (30/35) I: n=32; C: n=33	3 g omega-3 supplement	Placebo	Patients with psoriatic arthritis	PASI; BSA; DAS28-CRP
Tveit et al. (2020)	Parallel	26 weeks	64 (36/28) I: n=32; C: n=32	Herring roe oil: 2.6 g omega-3 supplement	Placebo	Patients with plaque psoriasis	PASI; BSA; DAS28-CRP; DLQI
Jarrett et al. (2018)	Parallel	12 months	65 (40/25) I: n=23; C: n=42	5 mg Vitamin D supplement	Placebo	Patients with psoriasis	PASI; DLQI
Greenberger et al. (2012)	Parallel	12 weeks	28 (19/9) I: n=17; C: n=11	alga Dunaliella bardawil. 9-cis B-carotene 30-40 mg/d	Placebo	Patients with plaque-type psoriasis	PASI; DLQI; Serum β-carotene
Narang et al. (2023)	Parallel	4 months	80 I: n=40; C: n=40	1.5 mg Vitamin D3	placebo	Patients with moderate chronic plaque psoriasis	PASI; DLQI
Narang et al. (2023)	Parallel	4 months	80 I: n=40; C: n=40	400 mg Vitamin E	placebo	Patients with moderate chronic plaque psoriasis	PASI; DLQI
Al-Oudah et	Parallel	12 weeks	24 (15/9)	100 mg CoQ10	Placebo	Patients with	PASI; DLQI

al. (2022)			I: n=13; C: n=11			persistent plaque psoriasis	
Nopriyati et a. (2023)	Parallel	12 weeks	30 (21/9) I: n=15; C: n=15	900 mg omega-3	Placebo	Patients with Mild-moderate chronic plaque psoriasis	PASI
Koch et al. (2008)	Parallel	8 weeks	44 (14/30) I: n=21; C: n=23	5.4 g DHA	Placebo	Patients with atopic eczema	SCORAD; Serum IgE
Panahi et al. (2012)	Parallel	4 weeks	96 (96/0) I: n=46; C: n=50	1 g curcumin	Placebo	Patients with chronic pruritic skin lesions	SCORAD; DLQI; SOD; CAT; GPx
Jaffary et al. (2015)	Parallel	4 months	65 (37/28) I: n=33; C: n=32	268 mg Vitamin E	Placebo	Mild to moderate AD patients	SCORAD
Mansour et al. (2020)	Parallel	3 months	86 (44/42) I: n=44; C: n=42	0.04 mg Vitamin D3 supplement	Placebo	Severe AD patients	EASI; Plasma 25(OH)D
Amestejani et al. (2012)	Parallel	60 days	60 I: n=30; C: n=30	0.04 mg Vitamin D supplement	Placebo	AD patients	SCORAD; Plasma 25(OH)D
Sara et al. (2023)	Parallel	14 days	40 (15/25) I: n=20; C: n=20	500 mg Vermint (Lumbricus rubellus Extract)	Placebo	Moderate to severe AD patients	SCORAD

Sánchez-Armendáriz et al. (2018)	Parallel	3 months	58<br>I: n=29;<br>C: n=29	0.125 mg vitamin D3 supplement	Placebo	Moderate AD patients	SCORAD; Plasma 25(OH)D
Stanley Xavier et al. (2020)	Parallel	2 months	72<br>I: n=36;<br>C: n=36	1.5 mg vitamin D supplement	Placebo	Parthenium dermatitis patients	EASI; DLQI; Plasma 25(OH)D
Panahi et al. (2012)	Parallel	4 weeks	80 (80/0)<br>I: n=40;<br>C: n=40	1 g curcumin: containing 500 mg curcuminoids plus 5 mg bioperinew.	Placebo	Chronic pruritic skin lesions patients	DLQI; IL-6 mRNA
Abe et al. (2022)	Parallel	8 weeks	30 (17/13)<br>I: n=15;<br>C: n=15	1 g polyphenol extracted from Fig leaf tea	Placebo	Mild AD patients	EASI
Zhou & Zhang (2023)	Parallel	12 weeks	120 (60/60)<br>I: n=60;<br>C: n=60	Polyphenol rich supplement	Placebo	Moderate to severe AD patients	SCORAD; DLQI; IL-6 mRNA
Patel et al. (2021)	Parallel	60 days	24<br>I: n=12;<br>C: n=12	500 mg Amalaki (Emblica offiicinalis) Extract	Placebo	Patients suffering from Acne vulgaris	Acne count; DLQI; Plasma TAC; Serum IgE

TEWL: Trans-Epidermal Water Loss; **SOD:** Superoxide dismutase; **CAT:** catalase; **GPx:** glutathione peroxidase; **IL-1β:** Interleukin-1 beta; **IL-6:** Interleukin-6; **TNF-α:** Tumor Necrosis Factor alpha; **IgE:** immunoglobulin E; **SCORAD:** SCORing Atopic Dermatitis; **DLQI:** Dermatology Life Quality Index; **PASI:** psoriasis area and severity index; **EASI:** Eczema Area Severity Index; **MDA:** Malondialdehyde; **MED:** Minimal Erythema Dose; **EPA:** Eicosapentaenoic Acid; **DHA:** Docosahexaenoic Acid; **LA:** Linoleic Acid; **EGCG:** Epigallocatechin Gallate; **BSA:** Body Surface Activity; **DAS28-CRP:** Disease Activity Score 28 - C-Reactive Protein; **TAC:** total antioxidant capacity; **25(OH)D:** 25-Hydroxyvitamin D.

Table S5 Summary and classification of the antioxidant-rich whole foods or supplement type

Antioxidant type	Detailed classification	Study type	Number of study (n)
Whole food	Fruits	Preclinical	1
	Nuts	Clinical	2
Supplements	Extracts	Preclinical	14
		Clinical	23
	Polyphenol	Preclinical	3
		Clinical	10
	Fatty acids	Preclinical	6
		Clinical	8
	Carotenoids	Preclinical	1
		Clinical	9
	Multi vitamins	Clinical	6
	Vitamin D	Clinical	6
	Vitamin E	Clinical	2
	Coenzyme Q10	Clinical	2
	Zinc	Clinical	1

Table S6 Effect of antioxidant-rich whole foods or supplements on preclinical skin health related biomarkers

**Outcome**	**Hedges'g**	**95%CI**
Anti-inflammatory marker		
SOD	1.56	[1.06; 2.05]
CAT	5.29	[2.46; 8.12]
GPx	4.16	[2.83; 5.50]
Oxidative stress marker		
IL-1β mRNA	-3.16	[-4.74; -1.58]
IL-6 mRNA	-2.67	[-3.52; -1.81]
TNF-α mRNA	-3.20	[-4.51; -1.88]
Immunological marker		
IgE	-2.55	[-4.43; -0.67]

SOD: Superoxide dismutase; **CAT:** catalase; **GPx:** glutathione peroxidase; **IL-1β:** Interleukin-1 beta; **IL-6:** Interleukin-6; **TNF-α:** Tumor Necrosis Factor alpha; **IgE:** immunoglobulin E.

(A) Hydration

Study	Hedges'g	95% CI	Weight (%)
**Subgroup = <12 weeks**			
Fujii et al. (2013)	1.10	[0.04; 2.16]	6.6
Ye et al. (2014 i)	0.84	[−0.08; 1.76]	7.2
Ye et al. (2014 ii)	1.87	[0.80; 2.93]	6.5
Ye et al. (2014 iii)	0.89	[−0.03; 1.82]	7.2
Hiragun et al. (2016 i)	2.06	[0.62; 3.50]	5.0
Hiragun et al. (2016 ii)	2.53	[0.96; 4.11]	4.5
Tamaru et al. (2020)	1.36	[0.18; 2.53]	6.0
Park et al. (2021i)	2.55	[0.97; 4.13]	4.5
Park et al. (2021ii)	5.32	[2.75; 7.89]	2.3
Park et al. (2023 i)	0.80	[−0.23; 1.82]	6.7
Park et al. (2023 ii)	2.23	[0.95; 3.50]	5.6
Park et al. (2023 iii)	3.01	[1.54; 4.49]	4.9
Lee et al. (2024 i)	0.83	[−0.20; 1.85]	6.7
Harauma et al. (2024 i)	1.50	[0.29; 2.72]	5.9
Lee et al. (2024 ii)	1.11	[0.05; 2.17]	6.6
Harauma et al. (2024 ii)	1.37	[0.15; 2.59]	5.8
Lee et al. (2024 iii)	2.26	[0.98; 3.55]	5.6
**Subtotal**	**1.57**	**[1.22; 1.92]**	**97.7**
Heterogeneity: I^2 = 42.4%, τ^2 = 0.1695, p = 0.0336			
**Subgroup = >=12 weeks**			
Geng et al. (2024)	6.50	[3.92; 9.07]	2.3
**Overall**	**1.75**	**[1.31; 2.20]**	**100.0**
Heterogeneity: I^2 = 59.6%, τ^2 = 0.4865, p = 0.0006			
Test for subgroup differences: χ^2_1 = 13.81, df = 1 (p = 0.0002)			

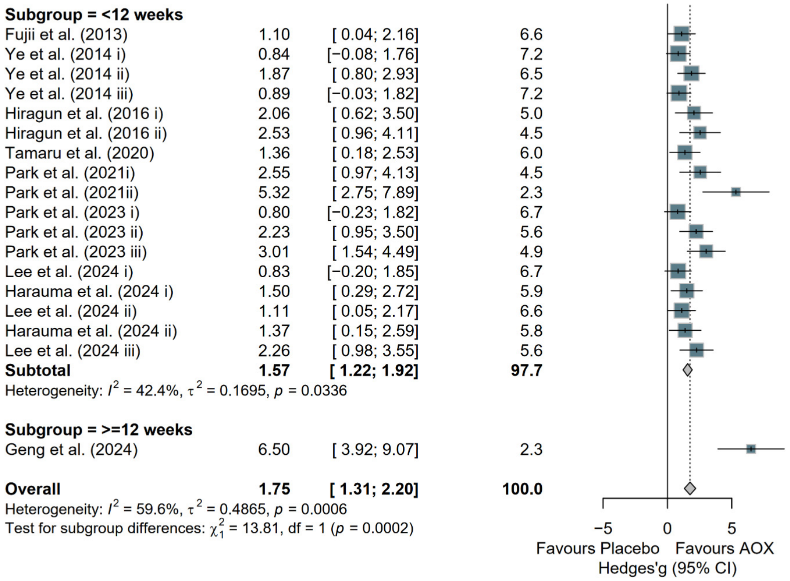

(B) Trans-epidermal water loss

Study	Hedges'g	95% CI	Weight (%)
**Subgroup = <12 weeks**			
Jeon et al. (2010)	−2.78	[−4.61; −0.95]	6.5
Fujii et al. (2013)	−2.90	[−4.35; −1.46]	7.0
Hiragun et al. (2016 i)	0.09	[−1.04; 1.22]	7.4
Hiragun et al. (2016 ii)	−1.30	[−2.56; −0.03]	7.3
Tamaru et al. (2020)	−0.65	[−1.73; 0.43]	7.5
Horie et al. (2024)	−8.90	[−12.92; −4.88]	3.6
Lee et al (2024 i)	−1.08	[−2.14; −0.02]	7.5
Harauma et al. (2024 i)	−1.06	[−2.21; 0.09]	7.4
Lee et al (2024 ii)	−1.11	[−2.17; −0.05]	7.5
Harauma et al. (2024 ii)	−1.20	[−2.39; −0.01]	7.4
Lee et al (2024 iii)	−2.09	[−3.33; −0.85]	7.3
**Subtotal**	**−1.48**	**[−2.10; −0.85]**	**76.5**
Heterogeneity: I^2 = 66.9%, τ^2 = 0.6333, p = 0.0008			
**Subgroup = >=12 weeks**			
Lee et al. (2018)	−9.72	[−13.71; −5.74]	3.7
Im et al. (2019)	−5.71	[−8.43; −2.99]	5.2
Kim et al. (2021 i)	−1.29	[−2.56; −0.03]	7.3
Kim et al. (2021 ii)	−1.03	[−2.25; 0.18]	7.3
**Subtotal**	**−4.11**	**[−7.95; −0.27]**	**23.5**
Heterogeneity: I^2 = 88.1%, τ^2 = 13.7953, p < 0.0001			
**Overall**	**−2.15**	**[−3.17; −1.13]**	**100.0**
Heterogeneity: I^2 = 75.6%, τ^2 = 3.3275, p < 0.0001			
Test for subgroup differences: χ^2_1 = 1.76, df = 1 (p = 0.1850)			

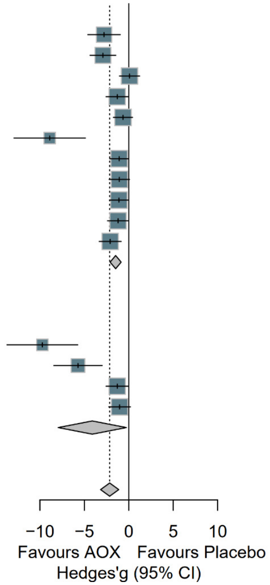

(C) Epidermal thickness

Study	Hedges'g	95% CI	Weight (%)
**Subgroup = <12 weeks**			
Jeon et al. (2010)	−2.72	[−4.53; −0.91]	3.1
Kim et al. (2019 i)	−1.40	[−2.68; −0.11]	3.4
Kim et al. (2019 ii)	−1.56	[−2.88; −0.24]	3.4
Kim et al. (2019 iii)	−1.89	[−3.29; −0.49]	3.4
Tamaru et al. (2020)	−6.08	[−8.71; −3.46]	2.5
Kim et al. (2020 i)	−3.60	[−5.24; −1.95]	3.2
Kim et al. (2020 ii)	−5.71	[−8.02; −3.40]	2.7
Kim et al. (2020 iii)	−6.29	[−8.80; −3.79]	2.6
Park et al. (2023 i)	−0.79	[−1.81; 0.24]	3.6
Park et al. (2023 ii)	−3.50	[−5.12; −1.89]	3.2
Park et al. (2023 iii)	−5.79	[−8.13; −3.45]	2.7
Lee et al (2024)	−4.02	[−5.78; −2.25]	3.1
Lee et al (2024 i)	−2.01	[−3.24; −0.79]	3.5
Harauma et al. (2024 i)	−3.68	[−5.44; −1.92]	3.1
Lee et al (2024 ii)	−2.90	[−4.34; −1.45]	3.3
Harauma et al. (2024 ii)	−2.24	[−3.65; −0.83]	3.4
Horie et al. (2024 vii)	−1.31	[−2.57; −0.04]	3.5
**Subtotal**	**−3.02**	**[−3.80; −2.24]**	**53.8**
Heterogeneity: I^2 = 73.1%, τ^2 = 1.9615, p < 0.0001			
**Subgroup = >=12 weeks**			
Viyoch et al. (2012 i)	−0.37	[−1.77; 1.04]	3.4
Viyoch et al. (2012 ii)	−0.56	[−1.99; 0.86]	3.4
Viyoch et al. (2012 iii)	−0.84	[−2.30; 0.62]	3.3
Viyoch et al. (2012 iv)	−1.39	[−2.98; 0.20]	3.2
Viyoch et al. (2012 v)	−1.05	[−2.56; 0.45]	3.3
Viyoch et al. (2012 vi)	−1.03	[−2.53; 0.47]	3.3
Song et al. (2013)	−1.59	[−3.06; −0.13]	3.3
Lee et al. (2018)	−4.16	[−6.11; −2.21]	3.0
Im et al. (2019)	−12.37	[−17.85; −6.89]	1.1
Kim et al. (2021 i)	0.06	[−1.07; 1.19]	3.5
Kim et al. (2021 ii)	−1.93	[−3.34; −0.53]	3.4
Geng et al. (2024)	−11.44	[−15.75; −7.14]	1.6
Park et al. (2024 i)	−0.14	[−1.12; 0.84]	3.6
Park et al. (2024 ii)	−1.57	[−2.71; −0.43]	3.5
Park et al. (2024 iii)	−3.96	[−5.71; −2.21]	3.1
**Subtotal**	**−2.17**	**[−3.45; −0.88]**	**46.2**
Heterogeneity: I^2 = 79.9%, τ^2 = 5.4492, p < 0.0001			
**Overall**	**−2.59**	**[−3.28; −1.89]**	**100.0**
Heterogeneity: I^2 = 79.3%, τ^2 = 3.2650, p < 0.0001			
Test for subgroup differences: χ^2_1 = 1.24, df = 1 (p = 0.2660)			

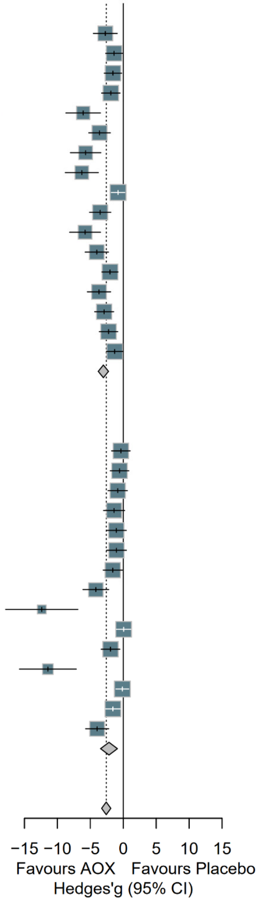

(D) Hyaluronic acid

Study	Hedges'g	95% CI	Weight (%)
**Subgroup = <12 weeks**			
Lee et al (2024 i)	0.83	[−0.20; 1.85]	18.1
Lee et al (2024 ii)	1.61	[0.47; 2.76]	17.5
Lee et al (2024 iii)	3.09	[1.59; 4.58]	15.5
**Subtotal**	**1.75**	**[0.51; 2.98]**	**51.1**
Heterogeneity: I^2 = 66.5%, τ^2 = 0.8068, p = 0.0505			
**Subgroup = >=12 weeks**			
Im et al. (2019)	26.40	[14.90; 37.90]	0.9
Kim et al. (2021 i)	0.19	[−0.95; 1.32]	17.5
Kim et al. (2021 ii)	2.43	[0.88; 3.98]	15.2
Geng et al. (2024)	3.26	[1.71; 4.80]	15.2
**Subtotal**	**6.88**	**[−3.51; 17.27]**	**48.9**
Heterogeneity: I^2 = 89.8%, τ^2 = 105.0525, p < 0.0001			
**Overall**	**2.05**	**[0.93; 3.17]**	**100.0**
Heterogeneity: I^2 = 83.0%, τ^2 = 1.5361, p < 0.0001			
Test for subgroup differences: χ^2_1 = 0.93, df = 1 (p = 0.3359)			

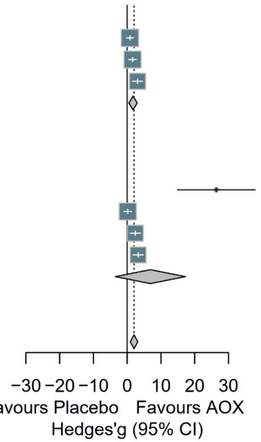

(E) Wrinkle formation

Study	Hedges'g	95% CI	Weight (%)
**Subgroup = < 12 weeks**			
Park et al. (2023 i)	−1.75	[−2.92; −0.58]	13.0
Park et al. (2023 ii)	−4.85	[−6.88; −2.82]	11.3
Park et al. (2023 iii)	−7.90	[−10.95; −4.84]	9.0
Lee et al (2024 i)	−1.83	[−3.01; −0.64]	13.0
Lee et al (2024 ii)	−4.37	[−6.25; −2.50]	11.6
Lee et al (2024 iii)	−8.86	[−12.25; −5.47]	8.3
**Subtotal**	**−4.62**	**[−6.90; −2.33]**	**66.0**
Heterogeneity: I^2 = 85.6%, τ^2 = 6.9010, p < 0.0001			
**Subgroup = >= 12 weeks**			
Kim et al. (2021 i)	−2.39	[−3.93; −0.86]	12.3
Kim et al. (2021 ii)	−5.50	[−8.14; −2.86]	9.9
Geng et al. (2024)	−4.10	[−5.90; −2.31]	11.8
**Subtotal**	**−3.76**	**[−5.47; −2.06]**	**34.0**
Heterogeneity: I^2 = 56.9%, τ^2 = 1.2809, p = 0.0980			
**Overall**	**−4.28**	**[−5.79; −2.76]**	**100.0**
Heterogeneity: I^2 = 79.9%, τ^2 = 4.2506, p < 0.0001			
Test for subgroup differences: χ^2_1 = 0.35, df = 1 (p = 0.5565)			

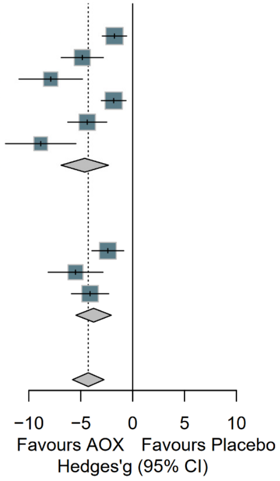

(F) IL-1 β mRNA

Study	Hedges'g	95% CI	Weight (%)
**Subgroup = >=12 weeks**			
Im et al. (2019)	−10.67	[−15.43; −5.91]	7.3
**Subgroup = < 12 weeks**			
Park et al. (2021 i)	−1.79	[−3.16; −0.42]	19.0
Park et al. (2021 ii)	−3.11	[−4.87; −1.35]	17.4
Park et al. (2023 i)	−1.23	[−2.31; −0.15]	20.1
Park et al. (2023 ii)	−3.21	[−4.74; −1.68]	18.4
Park et al. (2023 iii)	−3.70	[−5.38; −2.03]	17.8
**Subtotal**	**−2.48**	**[−3.46; −1.50]**	**92.7**
Heterogeneity: I^2 = 56.5%, τ^2 = 0.6859, p = 0.0564			
**Overall**	**−3.16**	**[−4.74; −1.58]**	**100.0**
Heterogeneity: I^2 = 76.1%, τ^2 = 2.9166, p = 0.0008			
Test for subgroup differences: χ^2_1 = 10.91, df = 1 (p = 0.0010)			

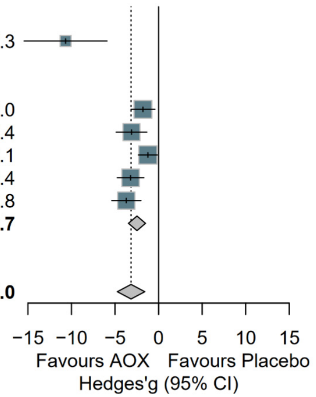

(G) IL-6 mRNA

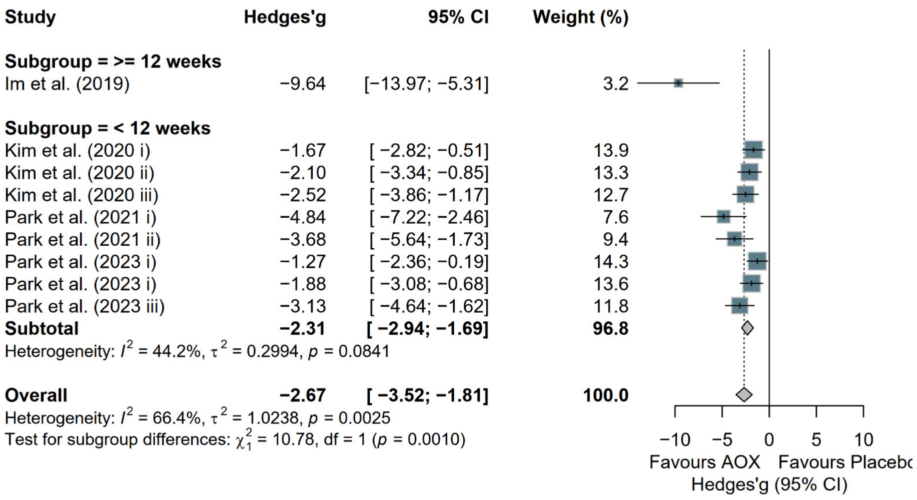

(H) TNF-α mRNA

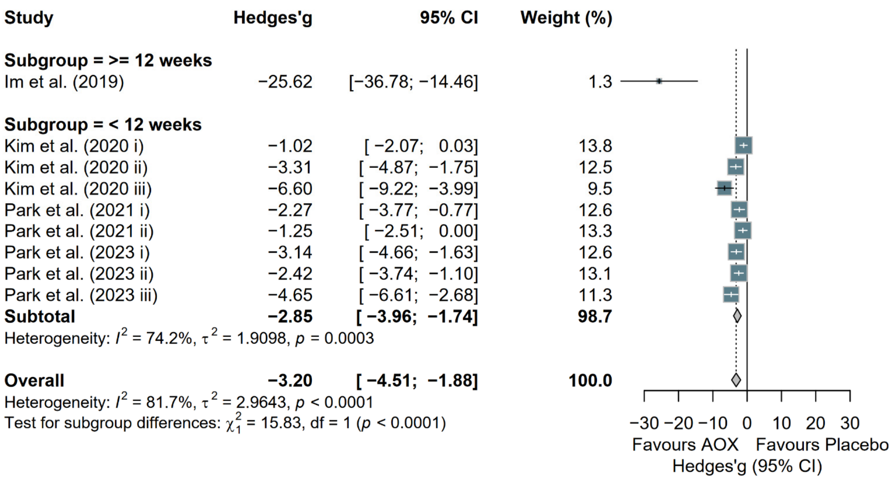

Figure S1. Forest plots of meta-analysis evaluating the effects of Antioxidant-rich whole foods or supplement (AOX) consumption on animal skin health, with subgroup analysis based on duration of study cohorts. The effect of AOX consumption on (A) Skin hydration in preclinical studies; (B) Trans-epidermal water loss in preclinical studies; (C) Epidermal thickness in preclinical studies; (D) Hyaluronic acid in preclinical studies; (E) Wrinkle formation in preclinical studies; (F) IL-1 β mRNA level in preclinical studies; (G) IL-6 mRNA level in preclinical studies; (H) TNF-α mRNA level in preclinical studies.

(A) Hydration

Study	MD	95% CI	Weight (%)
**Subgroup = >= 12 weeks**			
Heinrich et al. (2006)	7.00	[2.13; 11.87]	2.1
De Spirt et al. (2009 i)	3.00	[−3.42; 9.42]	1.6
De Spirt et al. (2009 ii)	2.00	[−3.58; 7.58]	1.9
Heinrich et al. (2011)	4.90	[0.63; 9.17]	2.3
De Sprit et al. (2012)	2.80	[−0.61; 6.21]	2.7
Jenkins et al. (2013 i)	−0.12	[−3.16; 2.91]	2.8
Jenkins et al. (2013 ii)	−0.84	[−2.64; 0.96]	3.3
Yoon et al. (2014)	−2.13	[−3.64; −0.62]	3.4
Mogollon et al. (2014)	−0.01	[−1.94; 1.91]	3.2
Bertuccelli et al. (2016)	27.79	[−9.97; 65.55]	0.1
Chiu et al. (2016)	1.78	[−0.37; 3.94]	3.2
Yoon et al. (2016)	−0.01	[−0.13; 0.11]	3.6
Tanaka et al. (2016)	8.76	[4.46; 13.06]	2.3
Kim et al. (2017)	1.22	[−1.94; 4.38]	2.8
Choi et al. (2018 i)	8.89	[4.76; 13.02]	2.4
Choi et al. (2018 ii)	7.05	[3.08; 11.02]	2.4
Uchiyama et al. (2019 i)	3.99	[1.22; 6.75]	2.9
Uchiyama et al. (2019 ii)	5.84	[3.33; 8.35]	3.0
Myung et al. (2020 i)	2.14	[1.07; 3.21]	3.5
Myung et al. (2020 ii)	2.45	[1.27; 3.64]	3.5
Nobile et al. (2021)	3.50	[0.27; 6.73]	2.7
Rybak et al. (2021)	−6.32	[−10.91; −1.72]	2.2
Li et al. (2021)	5.03	[1.69; 8.37]	2.7
Xie et al. (2022)	8.89	[−2.11; 19.89]	0.8
Tseng et al. (2022)	3.50	[0.70; 6.30]	2.9
Tumsutti et al. (2022)	−3.29	[−4.36; −2.22]	3.5
Ham et al. (2022)	1.41	[0.51; 2.31]	3.5
Rizzo et al. (2023)	0.37	[−0.18; 0.92]	3.6
Sato et al. (2024)	0.13	[−1.59; 1.85]	3.3
Sanjaya et al. (2024)	1.19	[−0.18; 2.57]	3.4
Handeland et al. (2024 i)	1.20	[0.33; 2.07]	3.5
Handeland et al. (2024 ii)	2.86	[1.88; 3.84]	3.5
**Subtotal**	**2.06**	**[0.99; 3.13]**	**88.5**
Heterogeneity: $I^2 = 87.6\%$, $\tau^2 = 7.1429$, $p < 0.0001$			
**Subgroup = <12 weeks**			
Ito et al. (2018)	2.97	[0.49; 5.45]	3.0
Hoshino et al. (2019)	−3.86	[−4.34; −3.38]	3.6
Kappler et al. (2022)	4.39	[0.00; 8.78]	2.3
Nobile et al. (2022)	7.50	[3.95; 11.05]	2.6
**Subtotal**	**2.51**	**[−2.41; 7.42]**	**11.5**
Heterogeneity: $I^2 = 96.1\%$, $\tau^2 = 22.8129$, $p < 0.0001$			
**Overall**	**2.12**	**[1.02; 3.21]**	**100.0**
Heterogeneity: $I^2 = 93.4\%$, $\tau^2 = 8.6789$, $p < 0.0001$			
Test for subgroup differences: $\chi^2_1 = 0.03$, df = 1 ($p = 0.8625$)			

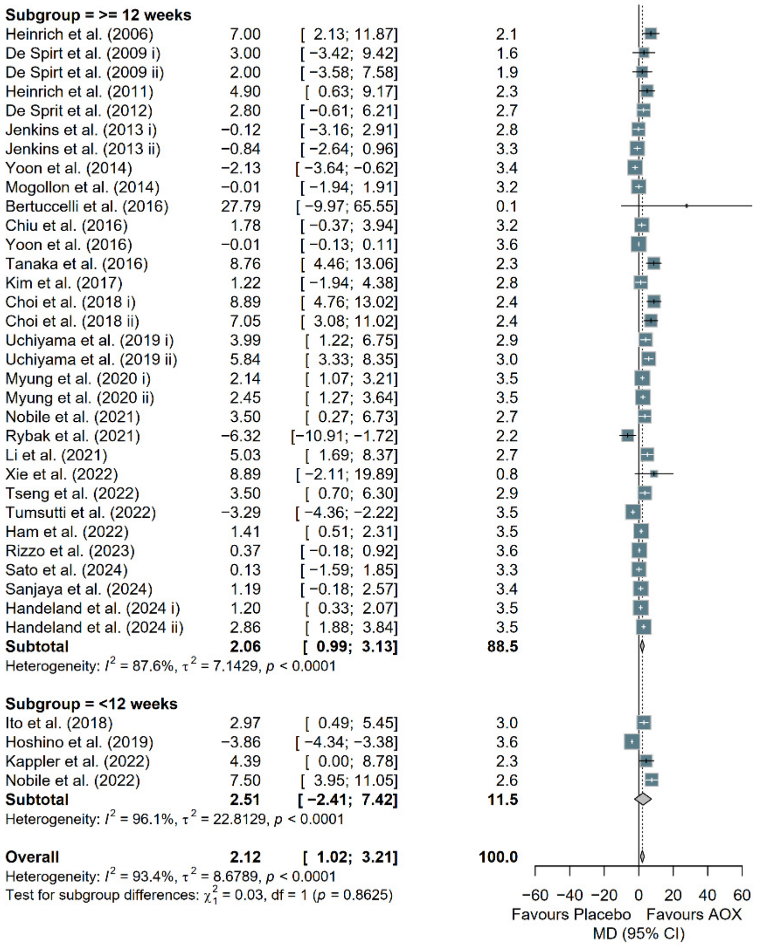

(B) Trans-epidermal water loss

Study	MD	95% CI	Weight (%)
**Subgroup = >= 12 weeks**			
De Sprit et al. (2012)	−1.00	[−2.56; 0.56]	3.8
Jenkins et al. (2013 i)	0.62	[−2.27; 3.52]	2.1
Jenkins et al. (2013 ii)	0.17	[−1.24; 1.58]	4.0
**Subtotal**	**−0.24**	**[−1.23; 0.74]**	**9.8**
Heterogeneity: $I^2 = 0\%$, $\tau^2 = 0$, $p = 0.4526$			
**Subgroup = 12 weeks**			
Heinrich et al. (2006 ii)	−2.10	[−5.72; 1.52]	1.5
De Spirt et al. (2009 i)	−2.40	[−5.00; 0.20]	2.4
De Spirt et al. (2009 ii)	−1.00	[−2.46; 0.46]	3.9
Heinrich et al. (2011 i)	−1.20	[−2.41; 0.01]	4.3
Yoon et al. (2014)	−1.71	[−2.07; −1.34]	5.4
Tanaka et al. (2016)	−1.08	[−1.76; −0.40]	5.1
Choi et al. (2018 i)	−3.28	[−6.85; 0.29]	1.6
Choi et al. (2018 ii)	−0.27	[−4.92; 4.38]	1.0
Uchiyama et al. (2019 i)	1.11	[−0.56; 2.78]	3.6
Uchiyama et al. (2019 ii)	0.34	[−1.04; 1.72]	4.0
Nobile et al. (2021)	−1.30	[−2.36; −0.24]	4.5
Xie et al. (2022)	−2.28	[−5.02; 0.46]	2.2
Tumsutti et al. (2022)	0.41	[−1.17; 1.99]	3.7
Ham et al. (2022)	−0.46	[−2.81; 1.89]	2.6
Tarshish et al. (2023)	0.54	[0.41; 0.67]	5.5
Sato et al. (2024)	−0.59	[−1.89; 0.71]	4.2
Sanjaya et al. (2024)	−0.52	[−1.50; 0.46]	4.7
Handeland et al. (2024 i)	−0.78	[−2.96; 1.40]	2.9
Handeland et al. (2024 ii)	−1.36	[−3.63; 0.91]	2.7
**Subtotal**	**−0.73**	**[−1.22; −0.24]**	**66.0**
Heterogeneity: $I^2 = 90\%$, $\tau^2 = 0.5438$, $p < 0.0001$			
**Subgroup = > 12 weeks**			
Puch et al. (2008)	0.42	[−0.23; 1.06]	5.1
Chiu et al. (2016)	−0.02	[−1.59; 1.55]	3.7
Yoon et al. (2016)	1.02	[−12.29; 14.33]	0.2
**Subtotal**	**0.35**	**[−0.24; 0.95]**	**9.0**
Heterogeneity: $I^2 = 0\%$, $\tau^2 = 0$, $p = 0.8767$			
**Subgroup = < 12 weeks**			
Maruki−Uchida et al. (2018)	2.33	[1.59; 3.07]	5.0
Hoshino et al. (2019)	−2.97	[−3.83; −2.12]	4.9
Kappler et al. (2022)	−3.54	[−6.47; −0.61]	2.1
Nobile et al. (2022)	−2.30	[−4.20; −0.40]	3.2
**Subtotal**	**−1.48**	**[−4.23; 1.27]**	**15.2**
Heterogeneity: $I^2 = 96.9\%$, $\tau^2 = 7.0827$, $p < 0.0001$			
**Overall**	**−0.68**	**[−1.21; −0.16]**	**100.0**
Heterogeneity: $I^2 = 90.0\%$, $\tau^2 = 1.3087$, $p < 0.0001$			
Test for subgroup differences: $\chi^2_3 = 8.36$, df = 3 ($p = 0.0390$)			

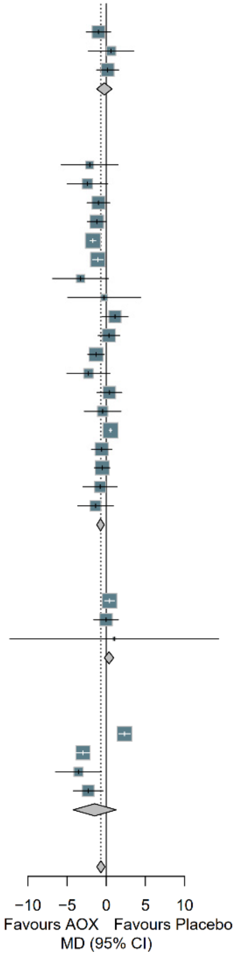

(C) Skin elasticity

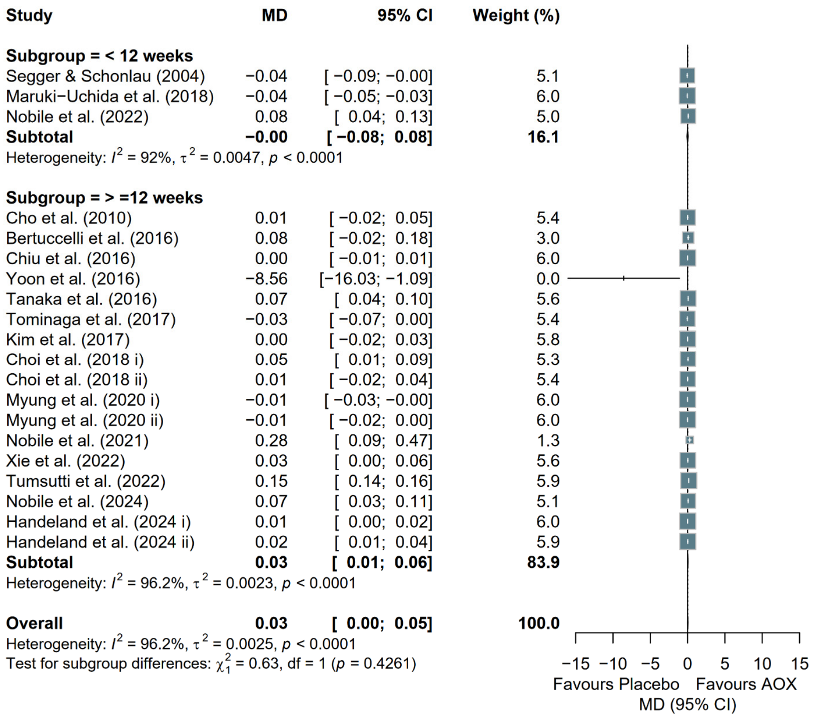

(D) SCORAD

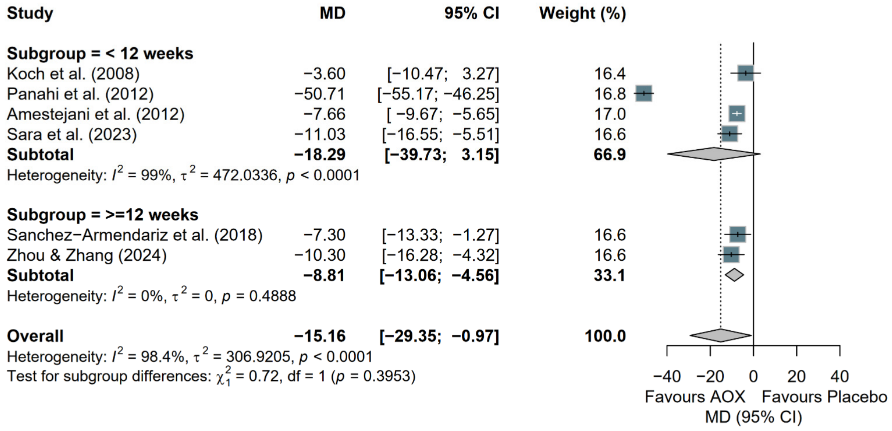

(E) Epidermal thickness

Study	MD	95% CI	Weight (%)
**Subgroup = >=12 weeks**			
Thom (2005)	0.33	[0.21; 0.45]	11.8
Heinrich et al. (2006)	0.14	[−0.01; 0.29]	10.1
Heinrich et al. (2006 i)	0.19	[−0.04; 0.42]	6.0
Heinrich et al. (2006 ii)	0.17	[−0.05; 0.40]	6.4
Heinrich et al. (2011)	0.02	[−0.05; 0.09]	15.7
De Sprit et al. (2012)	0.09	[−0.00; 0.18]	14.4
Uchiyama et al. (2019 i)	0.09	[0.04; 0.13]	17.7
Uchiyama et al. (2019 ii)	0.06	[0.02; 0.10]	17.8
**Subtotal**	**0.12**	**[0.05; 0.19]**	**100.0**
Heterogeneity: I^2 = 66.8%, τ^2 = 0.0064, p = 0.0036			
**Overall**	**0.12**	**[0.05; 0.19]**	**100.0**
Heterogeneity: I^2 = 66.8%, τ^2 = 0.0064, p = 0.0036			
Test for subgroup differences: χ^2_0 = 0.00, df = 0 (p = NA)			

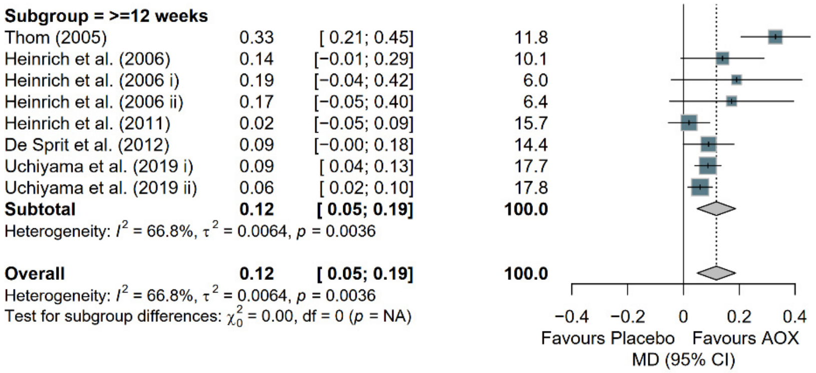

(F) Skin density

Study	MD	95% CI	Weight (%)
**Subgroup = >= 12 weeks**			
Heinrich et al. (2006 i)	0.90	[0.57; 1.23]	27.8
Heinrich et al. (2006 ii)	1.02	[0.67; 1.37]	26.9
Heinrich et al. (2011)	0.42	[0.36; 0.48]	35.9
De Sprit et al. (2012)	0.09	[−0.91; 1.09]	9.5
**Subtotal**	**0.68**	**[0.32; 1.04]**	**100.0**
Heterogeneity: I^2 = 84%, τ^2 = 0.0920, p = 0.0003			
**Overall**	**0.68**	**[0.32; 1.04]**	**100.0**
Heterogeneity: I^2 = 84.0%, τ^2 = 0.0920, p = 0.0003			
Test for subgroup differences: χ^2_0 = 0.00, df = 0 (p = NA)			

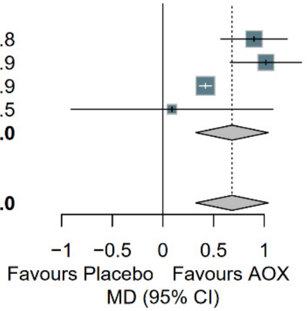

(G) DLQI

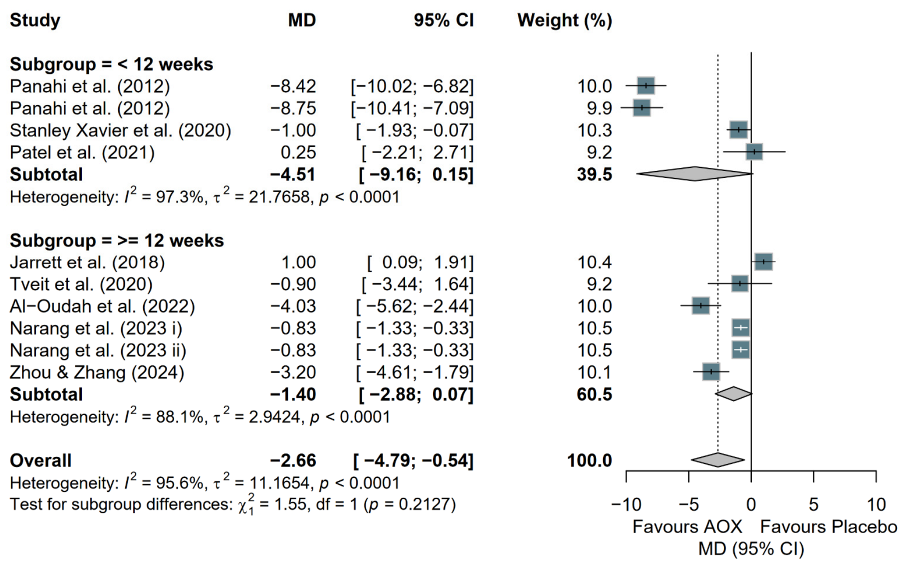

Figure S2. Forest plots of meta-analysis evaluating the effects of Antioxidant-rich whole foods or supplement (AOX) consumption on human skin health, with subgroup analysis based on duration of study cohorts. The effect of AOX consumption on (A) Skin hydration in clinical studies; (B) Trans-epidermal water loss in clinical studies; (C) Skin elasticity in clinical studies; (D) SCORAD in clinical studies; (E) Epidermal thickness in clinical studies; (F) Skin density in clnical studies; (G) DLQI in clinical studies.

Table S7. Sensitivity test of hydration in preclinical studies

Study_Omitted	Hedges'g	95% CI	I^2 (%)
Lee et al. 2024 i	1.83	[1.36; 2.3]	60%
Lee et al. 2024 ii	1.82	[1.34; 2.3]	61%
Lee et al. 2024 iii	1.74	[1.27; 2.21]	61%
Geng et al. 2024	1.57	[1.22; 1.92]	42%
Harauma et al. 2024 i	1.80	[1.31; 2.29]	62%
Harauma et al. 2024 ii	1.81	[1.32; 2.29]	62%
Hiragun et al. 2016 i	1.76	[1.28; 2.23]	62%
Hiragun et al. 2016 ii	1.72	[1.26; 2.17]	61%
Park et al. 2023 i	1.83	[1.36; 2.29]	60%
Park et al. 2023 ii	1.74	[1.27; 2.21]	61%
Park et al. 2023 iii	1.66	[1.24; 2.08]	58%
Park et al. 2021i	1.72	[1.26; 2.17]	61%
Park et al. 2021ii	1.62	[1.23; 2.01]	53%
Fujii et al. 2013	1.82	[1.34; 2.3]	61%
Tamaru et al. 2020	1.81	[1.32; 2.29]	62%
Ye et al. 2014 i	1.83	[1.37; 2.3]	60%
Ye et al. 2014 ii	1.78	[1.29; 2.27]	62%
Ye et al. 2014 iii	1.83	[1.36; 2.3]	60%
Overall	1.75	[1.31; 2.20]	60%

Table S8. Sensitivity test of TEWL in preclinical studies

Study Omitted	Hedges'g	95% CI	I^2 (%)
Lee et al 2024 i	-2.30	[-3.45; -1.14]	77%
Lee et al 2024 ii	-2.29	[-3.45; -1.14]	77%
Lee et al 2024 iii	-2.22	[-3.39; -1.06]	77%
Im et al. 2019	-1.77	[-2.56; -0.98]	73%
Lee et al. 2018	-1.62	[-2.25; -0.99]	68%
Kim et al. 2021 i	-2.28	[-3.44; -1.12]	77%
Kim et al. 2021 ii	-2.30	[-3.45; -1.15]	77%
Harauma et al. 2024 i	-2.30	[-3.45; -1.14]	77%
Harauma et al. 2024 ii	-2.29	[-3.44; -1.13]	77%
Hiragun et al. 2016 i	-2.32	[-3.38; -1.27]	74%
Hiragun et al. 2016 ii	-2.28	[-3.44; -1.12]	77%
Jeon et al. 2010	-2.16	[-3.29; -1.02]	76%
Horie et al. 2024	-1.69	[-2.39; -0.99]	70%
Fujii et al. 2013	-2.14	[-3.28; -1.01]	75%
Tamaru et al. 2020	-2.32	[-3.45; -1.19]	76%
Overall	-2.15	[-3.17; -1.13]	76%

Table S9. Sensitivity test of Dermatitis score in preclinical studies

Study Omitted	Hedges'g	95% CI	I^2 (%)
Lee et al. 2024 i	-2.78	[-4.76; -0.80]	90%
Lee et al. 2024 ii	-2.79	[-4.76; -0.82]	90%
Lee et al. 2024 iii	-2.83	[-4.74; -0.93]	89%
Kim et al. 2020 i	-2.49	[-4.51; -0.47]	90%
Kim et al. 2020 ii	-2.12	[-3.87; -0.38]	89%
Kim et al. 2020 iii	-2.06	[-3.73; -0.38]	89%
Kim et al. 2012 i	-2.34	[-4.27; -0.41]	89%
Kim et al. 2012 ii	-2.48	[-4.49; -0.46]	90%
Kim et al. 2012 iii	-1.88	[-3.33; -0.42]	89%
Zhou et al. 2016 i	-2.73	[-4.75; -0.72]	90%
Zhou et al. 2016 ii	-2.62	[-4.66; -0.57]	90%
Zhou et al. 2016 iii	-2.66	[-4.70; -0.62]	90%
Noli et al. 2007 i	-2.69	[-4.72; -0.66]	90%
Noli et al. 2007 ii	-2.74	[-4.75; -0.73]	90%
Noli et al. 2007 iii	-2.72	[-4.74; -0.70]	90%
Bensignor et al. 2008	-2.70	[-4.73; -0.66]	90%
Mueller et al. 2004 i	-2.72	[-4.74; -0.69]	90%
Mueller et al. 2004 ii	-2.70	[-4.73; -0.67]	90%
Overall	-2.55	[-4.43; -0.67]	90%

Table S10. Sensitivity test of Hyaluronic acid in preclinical studies

Study Omitted	Hedges'g	95% CI	I^2 (%)
Lee et al 2024 i	4.26	[-0.48; 9.01]	85%
Lee et al 2024 ii	4.23	[-0.77; 9.23]	86%
Lee et al 2024 iii	4.02	[-1.11; 9.15]	84%
Geng et al. 2024	3.98	[-1.14; 9.11]	84%
Im et al. 2019	1.80	[0.81; 2.80]	71%
Kim et al. 2021 i	2.35	[1.29; 3.41]	82%
Kim et al. 2021 ii	4.13	[-0.98; 9.23]	85%
Overall	2.05	[0.93; 3.17]	83%

Table S11. Sensitivity test of IgE level in preclinical studies

Study Omitted	Hedges'g	95% CI	I^2 (%)
Kim et al. 2020 i	-2.28	[-4.33; -0.22]	89%
Kim et al. 2020 ii	-2.09	[-3.95; -0.23]	89%
Kim et al. 2020 iii	-2.00	[-3.73; -0.26]	88%
Kim et al. 2012 i	-3.11	[-4.77; -1.45]	82%
Kim et al. 2012 ii	-2.75	[-4.92; -0.58]	91%
Kim et al. 2012 iii	-2.76	[-4.92; -0.59]	91%
Hiragun et al. 2016 i	-2.68	[-4.86; -0.49]	91%
Hiragun et al. 2016 ii	-2.75	[-4.91; -0.59]	91%
Overall	-2.55	[-4.43; -0.67]	90%

Table S12. Sensitivity test of Epidermal thickness in preclinical studies

Study Omitted	Hedges'g	95% CI	I^2 (%)
Lee et al 2024 i	-2.62	[-3.35; -1.89]	80%
Lee et al 2024 ii	-2.59	[-3.32; -1.86]	80%
Lee et al 2024	-2.54	[-3.26; -1.83]	79%
Geng et al. 2024	-2.39	[-3.02; -1.77]	77%
Park et al. 2024 i	-2.67	[-3.37; -1.97]	78%
Park et al. 2024 ii	-2.64	[-3.37; -1.91]	80%
Park et al. 2024 iii	-2.55	[-3.26; -1.83]	79%
Im et al. 2019	-2.44	[-3.09; -1.79]	78%
Lee et al. 2018	-2.54	[-3.25; -1.83]	79%
Kim et al. 2020 i	-2.56	[-3.28; -1.84]	79%
Kim et al. 2020 ii	-2.48	[-3.16; -1.79]	78%
Kim et al. 2020 iii	-2.46	[-3.13; -1.79]	78%
Kim et al. 2021 i	-2.67	[-3.37; -1.97]	78%
Kim et al. 2021 ii	-2.62	[-3.35; -1.89]	80%
Harauma et al. 2024 i	-2.56	[-3.28; -1.84]	79%
Harauma et al. 2024 ii	-2.61	[-3.34; -1.88]	80%
Jeon et al. 2010	-2.59	[-3.32; -1.87]	80%
Viyoch et al. 2012 i	-2.66	[-3.37; -1.95]	79%
Viyoch et al. 2012 ii	-2.66	[-3.37; -1.94]	80%
Viyoch et al. 2012 iii	-2.65	[-3.37; -1.93]	80%
Viyoch et al. 2012 iv	-2.64	[-3.36; -1.91]	80%
Viyoch et al. 2012 v	-2.65	[-3.37; -1.92]	80%
Viyoch et al. 2012 vi	-2.65	[-3.37; -1.92]	80%
Horie et al. 2024 vii	-2.64	[-3.37; -1.92]	80%
Park et al. 2023 i	-2.66	[-3.38; -1.94]	79%
Park et al. 2023 ii	-2.56	[-3.29; -1.84]	79%
Park et al. 2023 iii	-2.47	[-3.15; -1.79]	78%
Tamaru et al. 2020	-2.47	[-3.15; -1.79]	79%
Kim et al. 2019 i	-2.64	[-3.37; -1.91]	80%
Kim et al. 2019 ii	-2.63	[-3.36; -1.91]	80%
Kim et al. 2019 iii	-2.62	[-3.36; -1.89]	80%
Song et al. 2013	-2.63	[-3.36; -1.90]	80%
Overall	-2.59	[-3.28; -1.89]	79%

Table S13. Sensitivity test of Wrinkle formation in preclinical studies

Study Omitted	Hedges'g	95% CI	I^2 (%)
Lee et al 2024 i	-4.63	[-6.22; -3.04]	79%
Lee et al 2024 ii	-4.32	[-6.06; -2.57]	82%
Lee et al 2024 iii	-3.78	[-5.11; -2.46]	76%
Geng et al. 2024	-4.35	[-6.11; -2.60]	82%
Kim et al. 2021 i	-4.56	[-6.24; -2.89]	82%
Kim et al. 2021 ii	-4.17	[-5.84; -2.50]	81%
Park et al. 2023 i	-4.64	[-6.21; -3.06]	78%
Park et al. 2023 ii	-4.25	[-5.98; -2.52]	81%
Park et al. 2023 iii	-3.86	[-5.27; -2.45]	77%
Overall	-4.28	[-5.79; -2.76]	80%

Table S14. Sensitivity test of Pruritus score in preclinical studies

Study Omitted	Hedges'g	95% CI	I^2 (%)
Noli et al. (2007 i)	-0.11	[-0.62; 0.40]	0%
Noli et al. (2007 ii)	-0.23	[-0.75; 0.28]	0%
Noli et al. (2007 iii)	-0.12	[-0.62; 0.39]	0%
Bensignor et al. (2008)	-0.17	[-0.71; 0.36]	0%
Harvey et al. (1999 i)	-0.04	[-0.56; 0.48]	0%
Harvey et al. (1999 ii)	-0.23	[-0.75; 0.29]	0%
Overall	-0.15	[-0.62; 0.32]	0%

Table S15. Sensitivity test of SOD in preclinical studies

Study Omitted	Hedges'g	95% CI	I^2 (%)
Lee et al 2024 i	1.65	[1.16; 2.15]	54%
Lee et al 2024 ii	1.64	[1.14; 2.15]	56%
Lee et al 2024 iii	1.60	[1.09; 2.12]	60%
Park et al. 2024 i	1.56	[1.02; 2.10]	61%
Park et al. 2024 ii	1.57	[1.03; 2.11]	61%
Park et al. 2024 iii	1.55	[1.01; 2.09]	61%
Im et al. 2019	1.59	[1.08; 2.10]	60%
Park et al. 2023 i	1.60	[1.07; 2.13]	60%
Park et al. 2023 ii	1.56	[1.03; 2.09]	61%
Park et al. 2023 iii	1.51	[1.01; 2.01]	59%
Park et al. 2021 i	1.55	[1.02; 2.08]	61%
Park et al. 2021 ii	1.53	[1.00; 2.06]	61%
Ye et al. 2014 i	1.56	[1.02; 2.11]	61%
Ye et al. 2014 ii	1.53	[0.99; 2.07]	61%
Ye et al. 2014 iii	1.35	[1.04; 1.66]	0%
Overall	1.56	[1.06; 2.05]	58%

Table S16. Sensitivity test of CAT in preclinical studies

Study Omitted	Hedges'g	95% CI	I^2 (%)
Lee et al (2024 i)	5.65	[2.66; 8.64]	92%
Lee et al (2024 ii)	5.56	[2.51; 8.61]	92%
Lee et al (2024 iii)	5.37	[2.28; 8.46]	92%
Park et al. (2024 i)	5.65	[2.66; 8.64]	92%
Park et al. (2024 ii)	5.67	[2.69; 8.65]	92%
Park et al. (2024 iii)	5.62	[2.61; 8.64]	92%
Im et al. (2019)	5.28	[2.20; 8.35]	92%
Park et al. (2023 i)	5.53	[2.47; 8.59]	92%
Park et al. (2023 ii)	5.19	[2.13; 8.24]	92%
Park et al. (2023 iii)	5.10	[2.08; 8.11]	91%
Park et al. (2021i)	5.57	[2.53; 8.61]	92%
Park et al. (2021ii)	5.56	[2.51; 8.61]	92%
Ye et al. (2014 i)	5.08	[2.07; 8.10]	91%
Ye et al. (2014 ii)	4.39	[2.03; 6.76]	91%
Ye et al. (2014 iii)	4.21	[2.09; 6.34]	90%
Overall	5.29	[2.46; 8.12]	92%

Table S17. Sensitivity test of GPx in preclinical studies

Study Omitted	Hedges'g	95% CI	I^2 (%)
Lee et al (2024 i)	4.42	[2.97; 5.88]	80%
Lee et al (2024 ii)	4.37	[2.86; 5.87]	81%
Lee et al (2024 iii)	4.30	[2.76; 5.84]	81%
Park et al. (2023 i)	4.21	[2.67; 5.76]	81%
Park et al. (2023 ii)	4.28	[2.74; 5.82]	81%
Park et al. (2023 iii)	3.88	[2.55; 5.20]	77%
Ye et al. (2014 i)	4.45	[3.04; 5.86]	77%
Ye et al. (2014 ii)	3.60	[2.63; 4.57]	68%
Ye et al. (2014 iii)	4.00	[2.55; 5.46]	78%
Overall	4.16	[2.83; 5.50]	79%

Table S18. Sensitivity test of IL-1β in preclinical studies

Study Omitted	Hedges'g	95% CI	I^2 (%)
Im et al. (2019)	-2.48	[-3.46; -1.50]	56%
Park et al. (2023 i)	-3.65	[-5.41; -1.88]	71%
Park et al. (2023 ii)	-3.45	[-5.86; -1.04]	80%
Park et al. (2023 iii)	-3.32	[-5.66; -0.98]	78%
Park et al. (2021 i)	-3.69	[-5.89; -1.48]	80%
Park et al. (2021 ii)	-3.47	[-5.86; -1.08]	80%
Overall	-3.16	[-4.74; -1.58]	76%

Table S19. Sensitivity test of IL-6 in preclinical studies

Study Omitted	Hedges'g	95% CI	I^2 (%)
Im et al. (2019)	-2.31	[-2.94; -1.69]	44%
Kim et al. (2020 i)	-2.92	[-3.96; -1.87]	69%
Kim et al. (2020 ii)	-2.89	[-4.00; -1.78]	70%
Kim et al. (2020 iii)	-2.84	[-3.95; -1.72]	70%
Park et al. (2023 i)	-2.88	[-3.78; -1.98]	64%
Park et al. (2023 i)	-2.91	[-3.99; -1.83]	70%
Park et al. (2023 iii)	-2.71	[-3.76; -1.66]	69%
Park et al. (2021 i)	-2.34	[-3.01; -1.67]	63%
Park et al. (2021 ii)	-2.56	[-3.46; -1.66]	68%
Overall	-2.67	[-3.52; -1.81]	66%

Table S20. Sensitivity test of TNF-α in preclinical studies

Study Omitted	Hedges'g	95% CI	I^2 (%)
Im et al. (2019)	-2.85	[-3.96; -1.74]	74%
Kim et al. (2020 i)	-3.51	[-4.87; -2.15]	80%
Kim et al. (2020 ii)	-3.36	[-5.04; -1.68]	83%
Kim et al. (2020 iii)	-2.63	[-3.61; -1.65]	79%
Park et al. (2023 i)	-3.39	[-5.08; -1.69]	84%
Park et al. (2023 ii)	-3.49	[-5.18; -1.80]	84%
Park et al. (2023 iii)	-3.02	[-4.43; -1.61]	82%
Park et al. (2021 i)	-3.50	[-5.16; -1.83]	84%
Park et al. (2021 ii)	-3.53	[-4.99; -2.07]	82%
Overall	-3.20	[-4.51; -1.88]	82%

Table S21. Sensitivity test of hydration in clinical studies

Study Omitted	MD	95% CI	I^2 (%)
De Sprit et al. (2012)	2.11	[0.98; 3.23]	94%
Jenkins et al. (2013 i)	2.19	[1.07; 3.31]	94%
Jenkins et al. (2013 ii)	2.22	[1.10; 3.34]	94%
Yoon et al. (2014)	2.25	[1.16; 3.35]	94%
Handeland et al. (2024 i)	2.16	[1.03; 3.30]	94%
Handeland et al. (2024 ii)	2.10	[0.97; 3.24]	93%
Ito et al. (2018)	2.10	[0.97; 3.23]	94%
Choi et al. (2018 i)	1.92	[0.86; 2.98]	93%
Choi et al. (2018 ii)	1.98	[0.89; 3.07]	93%
Xie et al. (2022)	2.06	[0.97; 3.16]	94%
Tseng et al. (2022)	2.08	[0.96; 3.21]	94%
Bertuccelli et al. (2016)	2.09	[1.00; 3.19]	94%
Heinrich et al. (2011)	2.05	[0.94; 3.16]	94%
Heinrich et al. (2006)	2.00	[0.91; 3.10]	94%
Chiu et al. (2016)	2.14	[1.01; 3.27]	94%
Mogollon et al. (2014)	2.20	[1.07; 3.32]	94%
Tumsutti et al. (2022)	2.28	[1.22; 3.35]	93%
Sato et al. (2024)	2.19	[1.06; 3.32]	94%
Kappler et al. (2022)	2.07	[0.95; 3.18]	94%
Nobile et al. (2021)	2.09	[0.96; 3.21]	94%
Yoon et al. (2016)	2.20	[1.07; 3.33]	94%
Rybak et al. (2021)	2.27	[1.21; 3.33]	94%
De Spirt et al. (2009 i)	2.11	[0.99; 3.22]	94%
De Spirt et al. (2009 ii)	2.13	[1.01; 3.24]	94%
Kim et al. (2017)	2.15	[1.02; 3.28]	94%
Uchiyama et al. (2019 i)	2.07	[0.94; 3.19]	94%
Uchiyama et al. (2019 ii)	1.99	[0.89; 3.09]	93%
Ham et al. (2022)	2.16	[1.02; 3.29]	93%
Hoshino et al. (2019)	2.29	[1.25; 3.34]	88%
Li et al. (2021)	2.04	[0.92; 3.15]	93%
Sanjaya et al. (2024)	2.16	[1.03; 3.30]	94%
Nobile et al. (2022)	1.95	[0.87; 3.03]	93%
Tanaka et al. (2016)	1.93	[0.87; 3.00]	93%
Rizzo et al. (2023)	2.19	[1.06; 3.32]	94%
Myung et al. (2020 i)	2.13	[0.99; 3.27]	93%
Myung et al. (2020 ii)	2.12	[0.98; 3.25]	93%
Overall	2.12	[1.02; 3.21]	93%

Table S22. Sensitivity test of TEWL in clinical studies

Study Omitted	MD	95% CI	I^2 (%)
De Sprit et al. (2012)	-0.68	[-1.22; -0.13]	90%
Jenkins et al. (2013 i)	-0.71	[-1.25; -0.18]	90%
Jenkins et al. (2013 ii)	-0.72	[-1.27; -0.18]	90%
Yoon et al. (2014)	-0.63	[-1.17; -0.08]	84%
Handeland et al. (2024 i)	-0.68	[-1.23; -0.14]	90%
Handeland et al. (2024 ii)	-0.67	[-1.21; -0.13]	90%
Choi et al. (2018 i)	-0.64	[-1.17; -0.11]	90%
Choi et al. (2018 ii)	-0.69	[-1.22; -0.16]	90%
Xie et al. (2022)	-0.65	[-1.18; -0.11]	90%
Heinrich et al. (2011 i)	-0.66	[-1.21; -0.12]	90%
Heinrich et al. (2006 ii)	-0.66	[-1.20; -0.13]	90%
Chiu et al. (2016)	-0.71	[-1.26; -0.17]	90%
Tumsutti et al. (2022)	-0.73	[-1.27; -0.19]	90%
Sato et al. (2024)	-0.69	[-1.24; -0.14]	90%
Kappler et al. (2022)	-0.62	[-1.15; -0.10]	90%
Nobile et al. (2021)	-0.66	[-1.21; -0.11]	90%
Yoon et al. (2016)	-0.69	[-1.22; -0.16]	90%
De Spirt et al. (2009 i)	-0.64	[-1.18; -0.11]	90%
De Spirt et al. (2009 ii)	-0.68	[-1.22; -0.13]	90%
Uchiyama et al. (2019 i)	-0.75	[-1.28; -0.21]	90%
Uchiyama et al. (2019 ii)	-0.73	[-1.27; -0.18]	90%
Ham et al. (2022)	-0.69	[-1.24; -0.15]	90%
Hoshino et al. (2019)	-0.54	[-1.05; -0.04]	88%
Sanjaya et al. (2024)	-0.70	[-1.25; -0.14]	90%
Nobile et al. (2022)	-0.63	[-1.16; -0.09]	90%
Tanaka et al. (2016)	-0.67	[-1.22; -0.11]	90%
Puch et al. (2008)	-0.74	[-1.29; -0.20]	90%
Tarshish et al. (2023)	-0.76	[-1.30; -0.21]	83%
Maruki-Uchida et al. (2018)	-0.82	[-1.29; -0.35]	89%
Overall	-0.68	[-1.21; -0.16]	90%

Table S23. Sensitivity test of Epidermal thickness in clinical studies

Study Omitted	MD	95% CI	I^2 (%)
De Sprit et al. (2012)	0.13	[0.04; 0.21]	72%
Heinrich et al. (2006)	0.12	[0.04; 0.20]	71%
Heinrich et al. (2011)	0.14	[0.06; 0.21]	66%
Heinrich et al. (2006)	0.11	[0.04; 0.19]	70%
Heinrich et al. (2006)	0.12	[0.04; 0.19]	71%
Uchiyama et al. (2019)	0.13	[0.04; 0.21]	72%
Uchiyama et al. (2019)	0.13	[0.05; 0.21]	69%
Thom (2005)	0.07	[0.05; 0.10]	0%
Overall	0.12	[0.05; 0.19]	67%

Table S24. Sensitivity test of skin density in clinical studies

Study Omitted	MD	95% CI	I^2 (%)
De Sprit et al. (2012)	0.75	[0.36; 1.13]	89%
Heinrich et al. (2011)	0.91	[0.67; 1.14]	33%
Heinrich et al. (2006 i)	0.59	[0.10; 1.08]	82%
Heinrich et al. (2006 ii)	0.56	[0.16; 0.96]	76%
Overall	0.68	[0.32; 1.04]	84%

Table S25. Sensitivity test of skin sebum in clinical studies

Study Omitted	MD	95% CI	I^2 (%)
Xie et al. (2022)	-10.33	[-17.54; -3.12]	33%
Nobile et al. (2021)	-3.88	[-16.93; 9.17]	96%
Rybak et al. (2021)	0.75	[-6.28; 7.79]	18%
Li et al. (2021)	-5.35	[-15.79; 5.08]	96%
Overall	-4.59	[-14.05; 4.88]	94%

Table S26. Sensitivity test of skin elasticity in clinical studies

Study Omitted	MD	95% CI	I^2 (%)
Segger & Schonlau (2004)	0.03	[0.01; 0.05]	96%
Handeland et al. (2024 i)	0.03	[0.00; 0.05]	96%
Handeland et al. (2024 ii)	0.03	[0.00; 0.05]	96%
Choi et al. (2018 i)	0.03	[0.00; 0.05]	96%
Choi et al. (2018 ii)	0.03	[0.00; 0.05]	96%
Xie et al. (2022)	0.03	[0.00; 0.05]	96%
Cho et al. (2010)	0.03	[0.00; 0.05]	96%
Bertuccelli et al. (2016)	0.02	[0.00; 0.05]	96%
Maruki-Uchida et al. (2018)	0.03	[0.01; 0.06]	96%
Chiu et al. (2016)	0.03	[0.00; 0.05]	96%
Tumsutti et al. (2022)	0.02	[0.00; 0.03]	87%
Nobile et al. (2021)	0.02	[0.00; 0.05]	96%
Tominaga et al. (2017)	0.03	[0.01; 0.05]	96%
Yoon et al. (2016)	0.03	[0.00; 0.05]	96%
Kim et al. (2017)	0.03	[0.00; 0.05]	96%
Nobile et al. (2024)	0.02	[0.00; 0.05]	96%
Nobile et al. (2022)	0.02	[0.00; 0.05]	96%
Tanaka et al. (2016)	0.02	[0.00; 0.05]	96%
Myung et al. (2020 i)	0.03	[0.00; 0.05]	96%
Myung et al. (2020 ii)	0.03	[0.00; 0.05]	96%
Overall	0.03	[0.00; 0.05]	96%

Table S27. Sensitivity test of SCORAD in clinical studies

Study Omitted	MD	95% CI	I^2 (%)
Koch et al. (2008)	-17.43	[-33.86; -0.99]	99%
Panahi et al. (2012)	-7.91	[-9.58; -6.23]	0%
Amestejani et al. (2012)	-16.68	[-33.70; 0.34]	98%
Sara et al. (2023)	-15.97	[-33.21; 1.27]	99%
Sanchez-Armendariz et al. (2018)	-16.71	[-33.64; 0.22]	99%
Zhou & Zhang (2024)	-16.11	[-33.30; 1.07]	99%
Overall	-15.16	[-29.35; -0.97]	98%

Table S28. Sensitivity test of EASI in clinical studies

Study Omitted	MD	95% CI	I^2 (%)
Mansour et al. (2020)	-1.71	[-4.35; 0.93]	57%
Stanley Xavier et al. (2020)	1.75	[-8.02; 11.53]	91%
Abe et al. (2022)	3.04	[-4.06; 10.14]	83%
Overall	0.85	[-4.61; 6.30]	84%

Table S29. Sensitivity test of DLQI in clinical studies

Study Omitted	MD	95% CI	I^2 (%)
Panahi et al. (2012)	-2.02	[-3.94; -0.10]	94%
Stanley Xavier et al. (2020)	-2.86	[-5.21; -0.50]	96%
Panahi et al. (2012)	-1.99	[-3.85; -0.13]	94%
Patel et al. (2021)	-2.96	[-5.23; -0.69]	96%
Tveit et al. (2020)	-2.84	[-5.17; -0.52]	96%
Jarrett et al. (2018)	-3.09	[-5.29; -0.89]	95%
Narang et al. (2023 i)	-2.88	[-5.22; -0.53]	96%
Narang et al. (2023 ii)	-2.88	[-5.22; -0.53]	96%
Al-Oudah et al. (2022)	-2.51	[-4.87; -0.16]	96%
Zhou & Zhang (2024)	-2.60	[-4.98; -0.23]	96%
Overall	-2.66	[-4.79; -0.54]	96%

Table S30. Sensitivity test of PASI in clinical studies

Study Omitted	MD	95% CI	I^2 (%)
Tveit et al. (2020)	0.68	[-0.24; 1.59]	45%
Jarrett et al. (2018)	0.57	[-0.84; 1.98]	72%
Narang et al. (2023 i)	0.30	[-1.11; 1.71]	69%
Narang et al. (2023 ii)	0.21	[-1.13; 1.55]	67%
Al-Oudah et al. (2022)	-0.03	[-0.98; 0.91]	50%
Leite et al. (2022)	0.50	[-0.88; 1.89]	72%
Overall	0.34	[-0.78; 1.46]	65%

Table S31. Sensitivity test of MED in clinical studies

Study Omitted	MD	95% CI	I^2 (%)
Cho et al. (2010)	27.16	[4.26; 50.05]	86%
Yoon et al. (2016)	18.84	[-5.05; 42.73]	87%
Nobile et al. (2022)	25.03	[-0.69; 50.75]	89%
Ito et al. (2018)	25.57	[0.24; 50.89]	89%
Li et al. (2021)	17.35	[-4.05; 38.74]	88%
Rizwan et al. (2011)	25.11	[-0.59; 50.80]	89%
Baswan et al. (2020)	9.84	[-3.2; 22.88]	74%
Overall	21.56	[0.07; 43.04]	87%

Study	D1	D2	D3	D4
Lee (2024)	+	+	+	+
Geng (2024)	!	-	+	+
Park (2024)	!	-	+	+
Im (2019)	+	+	+	!
Lee (2018)	+	+	+	+
Kim (2020)	!	-	+	+
Kim (2021)	+	+	+	!
Kim (2012)	+	+	+	!
Harauma (2024)	!	-	+	-
Hiragun (2016)	+	-	+	+
Jeon (2010)	+	+	+	+
Zhou (2016)	+	!	+	+
Horie (2024)	+	+	+	+
Viyoch (2012)	+	+	+	!
Park (2023)	+	+	+	+
Park (2021)	+	+	+	+
Fujii (2013)	+	+	+	+
Tamaru (2020)	+	+	+	!
Noli (2007)	+	!	+	+
Bensignor (2008)	+	+	+	+
Harvey (1999)	+	!	+	+
Mueller (2004)	+	+	+	+
Kim (2019)	+	+	+	+
Song (2013)	+	+	+	+
Ye (2014)	+	+	+	+

+ Low risk
! Some concerns
- High risk

D1 Randomisation process
D2 Missing outcome data
D3 Measurement of the outcome
D4 Selection of the reported result

Figure S3. Risk of bias assessment of preclinical studies

(A)

Study	D1	D2	D3	D4	D5	Overall
De Sprit (2012)	+	+	+	+	+	+
Jenkins (2013)	+	+	+	+	!	!
Heinrich (2006)	!	+	+	+	+	!
Segger (2008)	!	+	+	+	+	!
Segger (2004)	!	+	+	+	!	!
Yoon (2014)	!	+	+	+	+	!
Handeland (2024)	+	+	+	+	+	+
Ito (2018)	+	+	+	+	+	+
Choi (2018)	+	+	+	+	+	+
Foolad (2019)	!	+	+	+	+	!
Xie (2022)	!	+	+	+	+	!
Cho (2010)	+	!	!	+	+	!
Puch (2008)	+	+	+	+	+	+
Tseng (2022)	+	+	+	+	+	+
Tarshish (2023)	!	+	+	!	+	!
Bertuccelli (2016)	+	+	+	!	+	!
Ulrike (2011)	+	+	+	+	+	+
Marruki-Uchida (2018)	+	+	+	+	+	+
Heinrich (2006)	+	!	+	+	+	!
Mogollon (2014)	+	+	+	+	+	+
Tumsutti (2022)	+	+	+	!	+	!
Chakkalakal (2023)	+	+	+	+	+	+
Sato (2024)	+	+	!	+	+	+
Kappler (2022)	+	+	+	+	+	+
Nobile (2021)	+	!	+	+	+	!
Tominaga (2017)	+	+	+	!	+	!
Yoon (2016)	+	!	+	+	+	!
Chakkalakal (2022)	+	+	+	+	+	+
Rybak (2021)	!	+	+	+	+	+
De Spirt (2009)	+	+	+	+	+	+
Kim (2017)	+	!	+	+	+	!
Žmitek (2016)	+	+	+	+	+	+
Uchiyama (2019)	+	+	+	!	+	!
Nobile (2024)	+	+	+	+	+	+
Ham (2022)	+	+	+	+	+	+
Rizzo (2023)	+	+	+	+	+	+
Hoshin (2018)	+	!	+	+	+	!
Li (2021)	!	+	+	+	+	!
Thom (2005)	+	+	+	+	+	+
Nobile (2022)	+	+	+	+	+	+
Lopresti (2024)	+	+	+	+	+	+
Tanaka (2017)	+	+	+	!	+	!

+ Low risk
! Some concerns
- High risk

D1 Randomisation process
D2 Deviations from the intended interventions
D3 Missing outcome data
D4 Measurement of the outcome
D5 Selection of the reported result

Myung (2020)	+	!	+	+	+	!
Rizwan (2011)	!	+	+	+	+	!
Baswan (2020)	+	+	+	!	+	!
Leite (2022)	+	+	+	+	+	+
Tveit (2020)	+	!	+	+	+	!
Jarrett (2017)	!	+	+	+	+	!
Greenberger (2013)	!	+	+	+	+	!
Narang (2023)	!	+	+	+	!	!
Al-Oudah (2022)	!	+	+	+	!	!
Nopriyati (2023)	!	+	+	+	!	!
Koch (2008)	+	+	+	+	+	+
Panahi (2011)	+	+	+	+	+	+
Jaffary (2015)	+	+	+	+	+	+
Mansour (2020)	+	+	+	+	+	+
Amestejani (2012)	+	+	+	+	!	!
Sara (2023)	!	+	+	+	+	!
Xavier (2019)	+	+	+	+	+	+
Sanchez-Armendariz (2018)	+	+	+	+	+	+
Panahi (2012)	+	+	+	+	+	+
Patel (2020)	!	+	+	!	+	!

(B)

Study	D1	DS	D2	D3	D4	D5	Overall
Zhao (2021)	+	!	+	+	+	+	!
Chiu (2016)	+	+	+	+	+	+	+

\+ Low risk

! Some concerns

\- High risk

D1 Randomisation process

DS Bias arising from period and carryover effects

D2 Deviations from the intended interventions

D3 Missing outcome data

D4 Measurement of the outcome

D5 Selection of the reported result

Figure S4. Risk of bias assessment of clinical studies (A) Parallel study; (B) Crossover study.

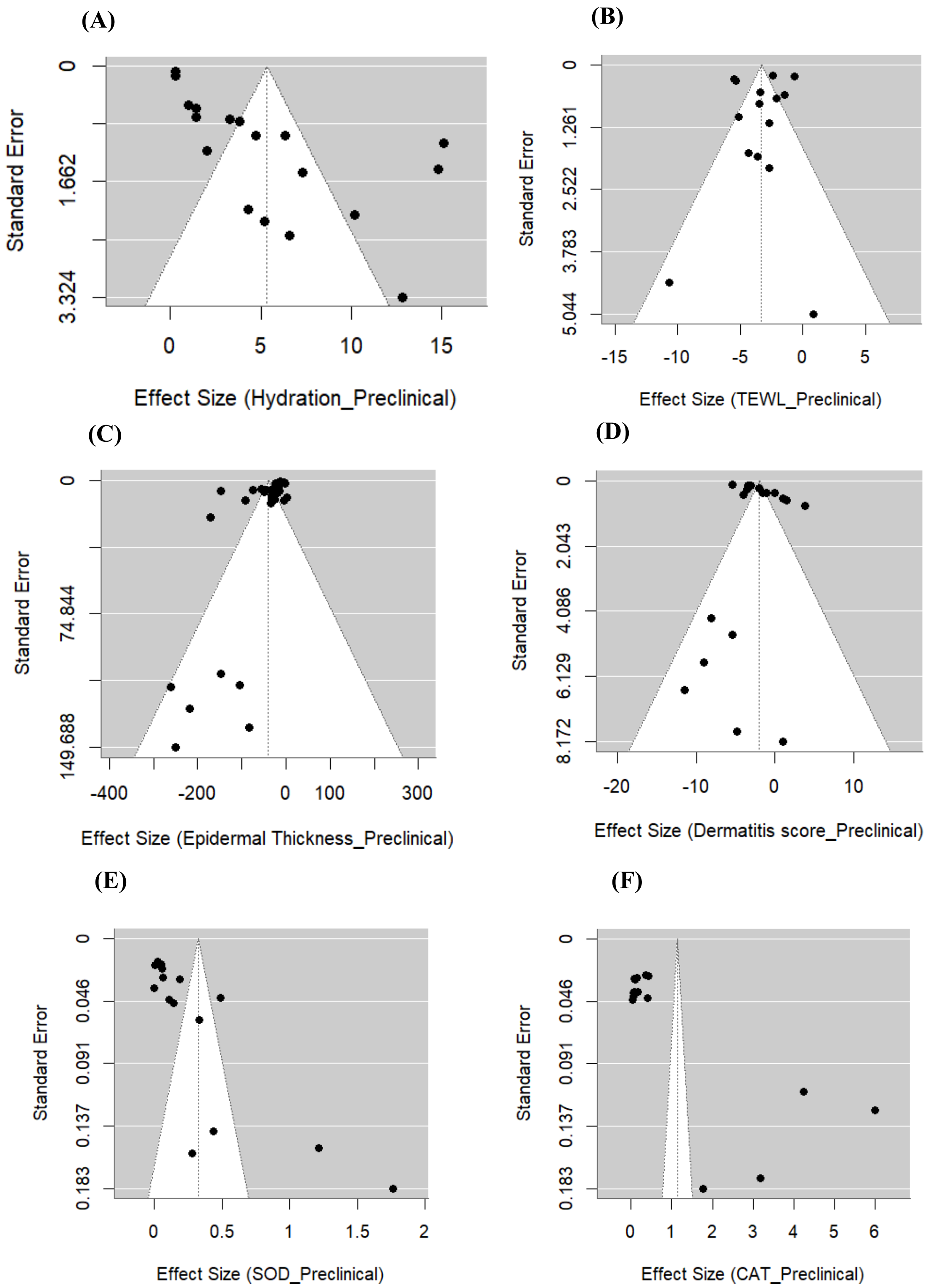

Figure S5. Preclinical studies, Funnel plot of **(A)** Hydration (Egger's test P < 0.0001); **(B)** Trans-epidermal water loss (Egger's test P = 0.5923); **(C)** Epidermal thickness (Egger's test P = 0.0012); **(D)** Dermatitis score (Egger's test P = 0.0616); **(E)** SOD (Egger's test P = 0.0009); **(F)** CAT (Egger's test P = 0.0024).

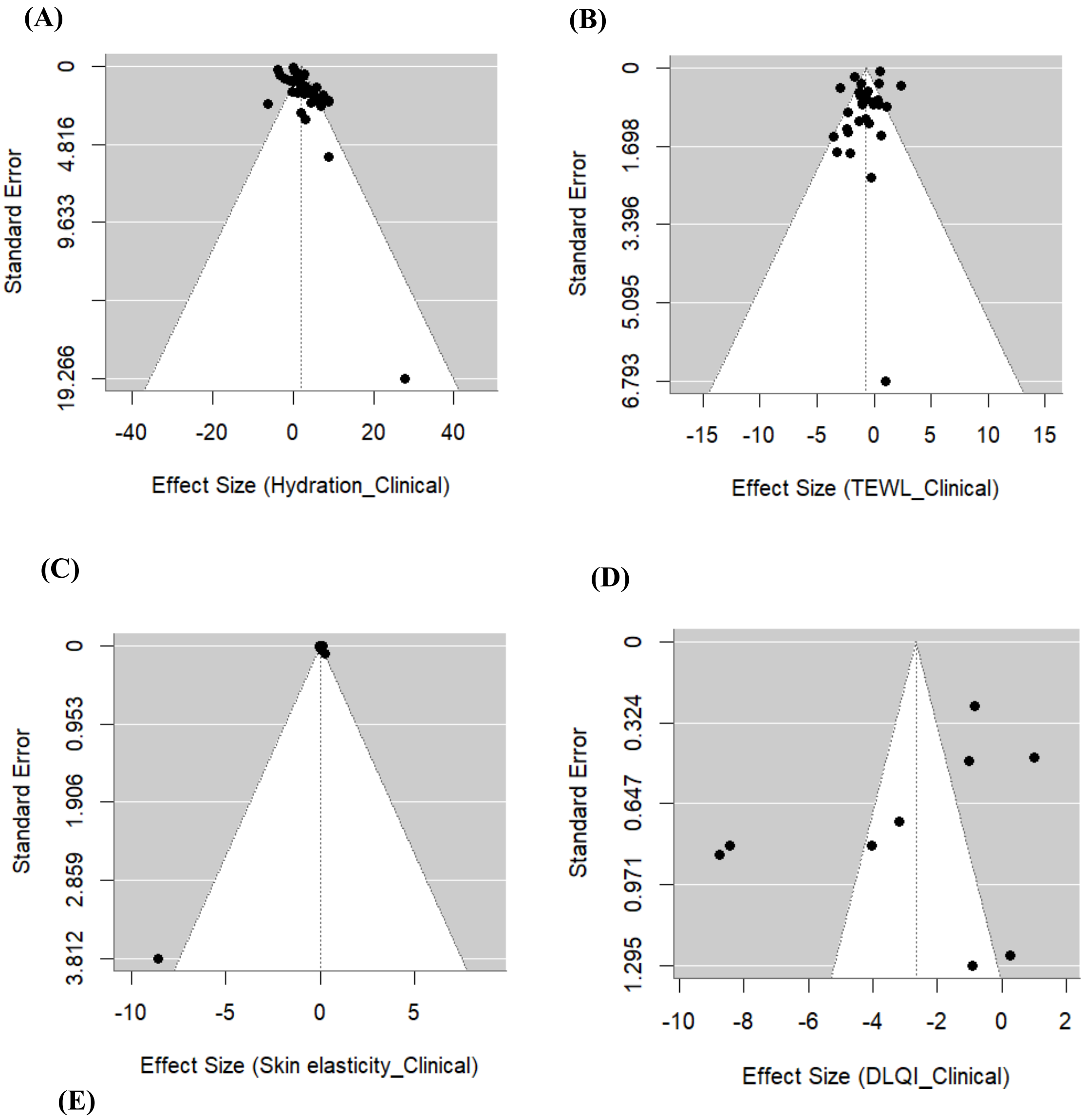

Figure S6. Clinical studies, Funnel plot of **(A)** Hydration (Egger's test P = 0.0261); **(B)** Trans-epidermal water loss (Egger's test P = 0.0216); **(C)** Skin elasticity (Egger's test P = 0.4338); **(D)** DLQI (Egger's test P = 0.1172).

Table S32. Bias-Aware (Trim and Fill) sensitivity test for hydration in preclinical studies

Parameter	Observed Value (Adjusted)	95% CI	p-value
**Pooled Mean Difference (MD)**	0.91 units	[-2.12; 3.93]	0.5570
**Total comparisons (k)**	27 (16 original + 9 added)	--	--
**Heterogeneity (I^2)**	96.9%	[96.2%; 97.5%]	< 0.0001
**Between-study Variance (tau^2)**	61.65	[37.47; 120.82]	--

Table S33. Bias-Aware (Trim and Fill) sensitivity test for TEWL in preclinical studies

Parameter	Observed Value (Adjusted)	95% CI	p-value
**Pooled Mean Difference (MD)**	-1.98 units	[-3.20; -0.77]	0.0014
**Total comparisons (k)**	21 (15 original + 6 added)	--	--
**Heterogeneity (I^2)**	97.1%	[96.3%; 97.7%]	< 0.0001
**Between-study Variance (tau^2)**	6.18	[3.21; 19.01]	--

Table S34. Bias-Aware (Trim and Fill) sensitivity test for Epidermal thickness in preclinical trial

Parameter	Observed Value (Adjusted)	95% CI	p-value
**Pooled Mean Difference (MD)**	-14.44 units	[-33.94; 5.06]	0.1466
**Total comparisons (k)**	46 (32 original + 14 added)	--	--
**Heterogeneity (I^2)**	97.8%	[97.5%; 98.1%]	< 0.0001
**Between-study Variance (tau^2)**	3520.51	[2542.74; 9177.64]	--

Table S35. Bias-Aware (Trim and Fill) sensitivity test for Dermatitis score in preclinical trial

Parameter	Observed Value (Adjusted)	95% CI	p-value
**Pooled Mean Difference (MD)**	-3.15 units	[-5.01; -1.30]	0.0008
**Total comparisons (k)**	20 (18 original + 2 added)	--	--
**Heterogeneity (I^2)**	97.7%	[97.1%; 98.1%]	< 0.0001
**Between-study Variance (tau^2)**	13.82	[6.43; 29.96]	--

Table S36. Bias-Aware (Trim and Fill) sensitivity test for SOD levels in preclinical trial

**Parameter**	**Observed Value (Adjusted)**	**95% CI**	**p-value**
**Pooled Mean Difference (MD)**	0.06 units	[-0.22; 0.34]	0.6768
**Total comparisons (k)**	21 (15 original + 6 added)	--	--
**Heterogeneity (I^2)**	96.7%	[95.8%; 97.4%]	< 0.0001
**Between-study Variance (tau^2)**	0.43	[0.25; 0.97]	--

Table S37. Bias-Aware (Trim and Fill) sensitivity test for CAT levels in preclinical trial

**Parameter**	**Observed Value (Adjusted)**	**95% CI**	**p-value**
**Pooled Mean Difference (MD)**	1.14 units	[0.20; 2.08]	0.0176
**Total comparisons (k)**	15 (15 original + 0 added)	--	--
**Heterogeneity (I^2)**	99.6%	[99.6%; 99.7%]	< 0.0001
**Between-study Variance (tau^2)**	3.44	[1.84; 8.58]	--

Table S38. Bias-Aware (Trim and Fill) sensitivity test for hydration in clinical trial

Parameter	Observed Value (Adjusted)	95% CI	p-value
**Pooled Mean Difference (MD)**	0.14 units	[-1.19; 1.46]	0.8385
**Total Studies (k)**	50 (36 original + 14 added)	--	--
**Heterogeneity (I^2)**	92.7%	[91.1%; 94.0%]	< 0.0001
**Between-study Variance (tau^2)**	18.94	[13.23; 37.98]	--

Table S39. Bias-Aware (Trim and Fill) sensitivity test for TEWL in clinical trial

Parameter	Observed Value (Adjusted)	95% CI	p-value
**Pooled Mean Difference (MD)**	0.41 units	[-0.24; 1.05]	0.2135
**Total Studies (k)**	42 (29 original + 13 added)	--	--
**Heterogeneity (I^2)**	93.4%	[91.9%; 94.6%]	< 0.0001
**Between-study Variance (tau^2)**	3.52	[2.10; 6.56]	--

Table S40. Bias-Aware (Trim and Fill) sensitivity test for skin elasticity in clinical trial

Parameter	Observed Value (Adjusted)	95% CI	p-value
**Pooled Mean Difference (MD)**	0.01 units	[-0.02; 0.04]	0.6865
**Total Studies (k)**	24 (20 original + 4 added)	--	--
**Heterogeneity (I^2)**	97.5%	[97.0%; 98.0%]	< 0.0001
**Between-study Variance (tau^2)**	0.01	[0.00; 0.03]	--

Table S41. Bias-Aware (Trim and Fill) sensitivity test for DLQI in clinical trial

Parameter	Observed Value (Adjusted)	95% CI	p-value
**Pooled Mean Difference (MD)**	-1.83 units	[-4.36; 0.69]	0.1549
**Total Studies (k)**	11 (10 original + 1 added)	--	--
**Heterogeneity (I^2)**	96.5%	[95.1%; 97.5%]	< 0.0001
**Between-study Variance (tau^2)**	17.65	[8.29; 55.98]	--

Table S42. Measurement Units and Statistical Models for Skin Outcomes in clinical studies.

Outcome	Common Units	Analysis Type	Justification
Hydration	Corneometer units	Mean Difference (MD)	Consistent units across studies
TEWL	$g/h/m^2$	Mean Difference (MD)	Standardized measurement protocol
Epidermal thickness	mm (micrometer)	Mean Difference (MD)	Consistent units across studies
Skin density	mm^2 (square micrometer)	Mean Difference (MD)	Consistent units across studies
MED	mJ/cm^2	Mean Difference (MD)	Consistent units across studies
Sebum	$\mu g/cm^2$	Mean Difference (MD)	Consistent units across studies
Skin elasticity	Ua/Uf	Mean Difference (MD)	Standardized measurement protocol
SCORAD	Score	Mean Difference (MD)	Standardized measurement protocol
DLQI	Score	Mean Difference (MD)	Standardized measurement protocol
PASI	Score	Mean Difference (MD)	Standardized measurement protocol
EASI	Score	Mean Difference (MD)	Standardized measurement protocol
